# Effect of Supplementation of Antioxidant Lipids Synthetized by Enzymatic Acidolysis with EPA/DHA Concentrate and Maqui (*Aristotelia chilensis* (Mol.) Stuntz) Seed Oil for Mitigating High-Fat Diet-Induced Obesity and Metabolic Disorders in Mice

**DOI:** 10.3390/antiox14070790

**Published:** 2025-06-26

**Authors:** Benjamín Claria, Alejandra Espinosa, Alicia Rodríguez, María Elsa Pando, Gretel Dovale-Rosabal, Nalda Romero, Katherynne Mayorga, Evelyn Tapia, Jenifer Saez, Melissa Tsuchida, Karla Vásquez, Rodrigo Valenzuela, Álvaro Pérez, Patricio Díaz, Santiago P. Aubourg

**Affiliations:** 1 Department of Food Science and Chemical Technology, Faculty of Chemical and Pharmaceutical Sciences, University of Chile, Dr. Carlos Lorca Tobar 964, Santiago 8380494, Chile; benjamin.claria@ug.uchile.cl (B.C.); gretel.dovale@ug.uchile.cl (G.D.-R.); nromero@uchile.cl (N.R.); katherynne.mayorga@ug.uchile.cl (K.M.); evelyn.tapia.l@ug.uchile.cl (E.T.); jenifer.saez@ug.uchile.cl (J.S.); melissa.tsuchida@ug.uchile.cl (M.T.); 2 Department of Medical Technology, Faculty of Medicine, University of Chile, Santiago 8380000, Chile; ealejand@uchile.cl (A.E.); patricio.diaz.c@ug.uchile.cl (P.D.); 3 School of Medicine, Faculty of Medicine, Universidad de Valparaíso, San Felipe 2172972, Chile; 4 Center of Interdisciplinary Biomedical and Engineering Research for Health, Universidad de Valparaíso, San Felipe 2172972, Chile; 5 Department of Nutrition, Faculty of Medicine, University of Chile, Santiago 8380000, Chile; pandosanmartin@uchile.cl (M.E.P.); ksvasquez@gmail.com (K.V.); rvalenzuelab@uchile.cl (R.V.); afperezb@uchile.cl (Á.P.); 6 Department of Food Technology, Marine Research Institute (CSIC), Eduardo Cabello 6, 36208 Vigo, Spain; saubourg@iim.csic.es

**Keywords:** obesity, metabolic syndrome, antioxidant lipids, EPA/DHA–omega-3, cold-pressed maqui seed oil, HFD male mice model, obesity-related biological parameters

## Abstract

Bioactive compounds have shown significant potential in the management of obesity and metabolic syndrome (MetS). This study investigates the effects of antioxidant lipids (ALω-3), synthetized through enzymatic acidolysis using non-specific lipase B from *Candida antarctica* under supercritical CO_2_ conditions. These lipids were derived from a concentrate of rainbow trout (*Oncorhynchus mykiss*) belly oil, rich in long-chain polyunsaturated omega-3 fatty acids (LCPUFAn-3), and cold-pressed maqui seed oil (MO, *Aristotelia chilensis* (Mol.) Stuntz). Their effects were then evaluated in a murine high-fat diet (HFD) model. The fatty acid profile, tocopherol and tocotrienol content, and thin-layer chromatography of ALω-3 were analyzed. After 8 weeks on an HFD, male C57BL/6 mice were divided into four groups and switched to a control diet (CD) with the following supplements for 3 weeks: Glycerol (G), commercial marine Omega-3 (CMω-3), a mixture of LCPUFAn-3 concentrate + MO (Mω-3), or ALω-3. The total body and organ weights, serum markers, and liver and visceral fat pro-inflammatory marker expression levels were assessed. ALω-3 contained 13.4% oleic, 33.9% linoleic, 6.3% α-linolenic, 10.7% eicosapentaenoic, and 16.2% docosahexaenoic fatty acids. The β, γ, δ-tocopherol, and β, γ-tocotrienol values were 22.9 ± 1.4, 24.9 ± 0.2, 6.8 ± 0.7, 22.9 ± 1.7, and 22.4 ± 4.7 mg·kg^−1^, respectively, with α-tocopherol detected in traces. ALω-3 supplementation increased serum Trolox equivalent capacity, significantly reduced serum GPT levels (*p* < 0.01), and enhanced postprandial glucose tolerance (*p* < 0.001), although it did not alter insulin resistance (HOMA-IR). These findings indicate ALω-3′s potential for mitigating the glucose intolerance, liver damage, and oxidative stress associated with obesity and MetS, highlighting the need for additional research to explore its potential health benefits.

## 1. Introduction

Metabolic syndrome (MetS) is defined as a cluster of metabolic abnormalities—including abdominal obesity, dyslipidemia, hypertension, and insulin resistance—which together increase the risk of cardiovascular disease and type 2 diabetes (T2D) [1]. The prevalence of MetS has been rising steadily worldwide, closely mirroring the global increase in obesity rates [2]. This trend is largely attributed to a shift in dietary patterns, marked by higher consumption of calorie-dense foods that are rich in fats and sugars, combined with more sedentary lifestyles [3]. In recent years, there has been growing recognition of the need to better define other metabolic diseases, such as metabolic dysfunction-associated steatotic liver disease (MASLD), previously referred to as non-alcoholic fatty liver disease (NAFLD) [4]. Notably, chronic inflammation and oxidative stress play a pivotal role in the pathophysiology of MetS and its comorbidities, contributing to their onset, progression, and severity [5].

To address these underlying mechanisms, bioactive compounds (BCs) are being actively studied as potential therapeutic agents for MetS. These compounds exert pharmacological effects in humans, playing key roles in cellular defense and signaling pathways [6,7]. Their use offers several advantages, including widespread availability, safety, and minimal side effects [6,7]. An example of such BCs is long-chain polyunsaturated omega-3 fatty acids (LCPUFAn-3), which are primarily found in oily fish (eicosapentaenoic acid, C20:5n-3, EPA; docosahexaenoic acid, C22:6n-3, DHA) [8]. Meanwhile, certain plant-based sources, such as seeds and nuts, are known to provide linoleic acid (LA, C18:2n-6) and, to a lesser extent, α-linolenic acid (ALA, C18:3n-3) [9]. In a study by Sanchez et al. [10], the seed oil of maqui (*Aristotelia chilensis* Mol. Stuntz), a native Chilean tree, was reported to contain approximately 50.4 g of LA per 100 g of total fatty acids, along with 1.7% ALA.

Both EPA and DHA are especially recognized for their beneficial effects in reducing the risk of cardiovascular diseases, mitigating inflammation, and lowering blood pressure [8]. These health benefits associated with EPA and DHA have generated significant interest in extracting and concentrating these LCPUFAn-3 from marine organisms, which are widely recognized as the most important natural sources of these compounds [11].

Despite the therapeutic benefits of bioactive compounds, a significant challenge lies in their bioavailability and stability. Many of these compounds—such as LCPUFAn-3—are susceptible to factors such as oxidation, which can reduce their effectiveness in the body [12]. Antioxidants such as tocopherols and tocotrienols—naturally present in nuts, seeds, and vegetable oils—help counteract this issue by scavenging peroxyl radicals and reactive nitrogen species [13]. Maqui seed oil (MO) has been reported to be rich in antioxidant compounds, including tocopherols and tocotrienols [10], which may contribute to the oxidative protection of LCPUFAn-3, improving their stability and functional delivery in vivo. In addition to their antioxidant activity, tocopherols and tocotrienols have also been shown to modulate key signaling pathways and inflammatory mediators involved in metabolic regulation and oxidative stress [14].

**Figure 1:**
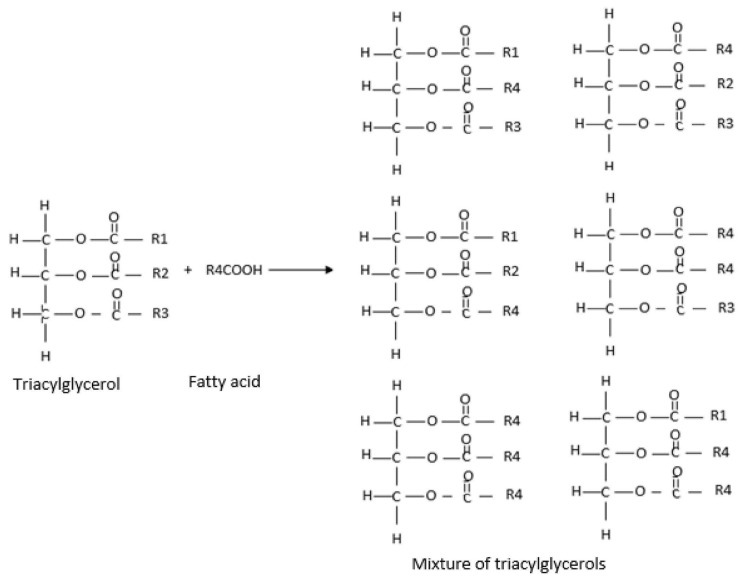
Schematic diagram showing mechanism of acidolysis, Sivakanthan et al. [15].

One method to synthesize bioactive lipids is through transesterification reactions, with this study specifically focusing on enzymatic acidolysis [15]. Enzymatic acidolysis involves the transfer of an acyl group between an acid and an ester, catalyzed by lipase, and serves as an effective means of incorporating new fatty acids into a TAG (Figure 1) [15].

Combining MO-derived antioxidants with EPA and DHA may offer a synergistic strategy to enhance the biological activity of omega-3 fatty acids. Emerging evidence suggests that tocopherols and tocotrienols can potentiate the anti-inflammatory and metabolic effects of omega-3s by preserving their integrity and facilitating their incorporation into cellular membranes, thereby amplifying their impact on inflammation resolution, lipid metabolism, and oxidative stress modulation [16,17]—all key factors in the progression of MetS and MASLD [2,5]. Based on this, the synthesis of antioxidant bioactive lipids through enzymatic acidolysis using marine EPA/DHA concentrates and MO could result in a novel lipid matrix with improved functional properties for the prevention or mitigation of these metabolic disorders.

A recently explored medium for the synthesis of bioactive lipids is supercritical carbon dioxide (SCCO_2_). This methodology has recently attracted a great deal of attention as an environmentally friendly tool for various purposes [18,19]. Due to its unique viscosity, fluidity, and solvation characteristics, SCCO_2_ enhances mass transfer during enzymatic reactions [20]. Additionally, it offers several advantages, including its designation as GRAS (Generally Recognized As Safe), low cost, and the ability to be easily separated from the reaction medium through depressurization [19].

The aim of this work was to generate antioxidant lipids (ALω-3) through enzymatic acidolysis, including EPA/DHA from concentrated rainbow trout (*Oncorhynchus mykiss*) belly oil (RTBO)—rich in LCPUFAn-3—and tocopherol and tocotrienol compounds from cold-pressed maqui seed oil (*Aristotelia chilensis* (Mol.) Stuntz) (MO), using non-specific lipase B from *Candida antarctica* under SCCO_2_ conditions. The study also aimed to evaluate its effects on reversing the early stages of metabolic alterations, inflammation, and oxidative stress associated with MetS and MASLD in mice fed a high-fat diet (HFD).

## 2. Materials and Methods

### 2.1. Reagents

Rainbow trout (*Oncorhynchus mykiss*) bellies were obtained from the aquaculture company Salmones Antártica S.A. (Puerto Montt, Chile); the fish had been raised and processed at their plant in Chiloé, Chile [21]. Cold-pressed maqui (*Aristotelia chilensis* (Mol.) Stuntz) seed oil (MO) was purchased from the company De Castañas y Amores (Santiago, Chile). Both items were preserved at −80 °C and protected from light [20,21]. Commercial marine Omega 3 (CMω-3) was obtained from a local pharmacy. Immobilized Lipozyme^®^ 435 lipase from *Candida antarctica* was used for enzymatic acidolysis; the enzyme was immobilized on a non-compressible silica gel carrier (Novozymes A/S, Bagsværd, Denmark) and was obtained from the company Merck S.A. (Santiago, Chile).

Both the methyl tricosanoate internal standard and the gas–liquid chromatography reference standard GLC-463 used in the analyses were obtained from Nu-Chek-Prep, Inc. (Elysian, MN, USA). CO_2_, H_2_, N_2_—with a purity of more than 99.95%—and zero air gas cylinders were purchased from GasLab-Linde (Santiago, Chile). α, β, γ, δ-tocopherol, and β, γ-tocotrienol standards were purchased from CalbioChem Merck (Santiago, Chile). Serum insulin was measured using the Mouse Insulin Elisa Kit from Mercodia (Uppsala, Sweden), and blood glucose levels were measured using test strips from OneTouch^®^ Ultra^TM^ (Milpitas, CA, USA). Biochemical parameters in serum were measured using the KENSHIN-2 multi-reagent strips from Arkray (Kyoto, Japan). PCR was performed with the DNA-free^TM^ DNA extraction kit from Invitrogen^TM^ (Carlsbad, CA, USA), High Capacity cDNA Reverse Transcription Kit 4368814 from Applied Biosystems^TM^ (Foster City, CA, USA), and Brillant II SYBR Green qPCR Master Mix ST 600828 from Agilent Technologies (Santa Clara, CA, USA). Other chemical reagents were of analytical grade.

### 2.2. Extraction of Belly Oil from Rainbow Trout

Oil was extracted from the belly of the rainbow trout using a modified version of the Radin method [11,22,23]. A sample of 100 g of belly tissue, thawed overnight in the refrigerator, was weighed and then ground in a food processor. The tissue was mixed in a beaker with 1800 mL of a solvent solution consisting of hexane and isopropanol in a 3:2 (*v*/*v*) ratio. The mixture was then vacuum-filtered through Whatman No. 1 filter paper using a Büchner funnel, with the solid phase discarded. The filtered liquid phase was combined with 700 mL of 3% anhydrous Na_2_SO_4_ solution and stirred for 10 min with a magnetic stirrer. Afterward, the mixture was vacuum-filtered again, and the solid phase was discarded. The resulting solution was transferred to a separation funnel to separate the organic and aqueous phases. The organic phase, which contained the desired oil, was collected. To remove the solvent, the organic phase was evaporated using a rotary evaporator at 40 °C for about 2 h, or until complete solvent removal. The crude oil was then nitrogen-flushed and stored in an ultra-freezer at −80 °C until further use.

### 2.3. LCPUFAn-3 Concentrate Preparation

Concentrates from rainbow trout belly oil (RTBO) were prepared using the urea-complexation method, modified from Zuta et al. [11,23,24,25]. First, the fatty acids—obtained through saponifying RTBO according to the protocol described by Guil-Guerrero [25]—were combined with urea in a 7.2:1 ratio (urea to fatty acid (FA)) and 95% ethanol. The mixture was stirred at 200 rpm and heated to 21 °C until the urea dissolved, forming a clear homogeneous solution [26]. Subsequently, the urea–FA adducts were allowed to crystallize at −21 °C for 15.5 h, and the urea crystals were separated by filtration using Whatman No. 1 filter paper and a Büchner funnel. The non-urea-complex fraction was then diluted with 100 mL of distilled water, acidified to pH 4.5 with 6 N HCl, and extracted twice with 50 mL of hexane. The two hexane extracts were combined and dried over anhydrous sodium sulfate. The solvent was partially removed under reduced pressure using a rotary evaporator at 45 °C. The resulting LCPUFAn-3 concentrates were stored at −80 °C under a nitrogen atmosphere until further use.

### 2.4. Synthesis of Antioxidant Lipids

Antioxidant lipids (ALω-3) were obtained through enzymatic acidolysis using immobilized non-specific lipase B from *Candida antarctica* (Immobilized Lipozyme^®^ 435; 10% of the total weight of the substrate) on a mixture of concentrated RTBO—rich in LCPUFAn-3 EPA/DHA—and MO in a 70/30 (*w*/*w*) ratio (Mω-3). For this process, a high-pressure reactor, specifically the Speed SFE system model 7071 supercritical CO_2_ equipment (Speed^TM^ SFE, Applied Separation, Allentown, PA, USA), was employed. The reaction was carried out under supercritical CO_2_ conditions at 80 °C and 300 psi on a stainless-steel column with a capacity of 10 g of sample for 2 h of reaction time, followed by 2 h of extraction. The resulting ALω-3 was stored at −80 °C in PET (polyethylene terephthalate) bottles, protected from light and humidity, until further use.

### 2.5. Experimental Design Using Response Surface Methodology (RSM) for Enzymatic Acidolysis Under Supercritical CO_2_ Condition

For the experimental design, a Factorial 2^2 design was created using a response surface methodology (RSM), as described by Myers and Montgomery [27]. The following two independent variables were studied: supercritical CO_2_ temperature at 50, 65, and 80 °C; and supercritical CO_2_ pressure at 100, 200, and 300 bar. The reaction time was kept constant at 2 h for the enzymatic reaction and 2 h for extraction, as well as a 70/30 (*w*/*w*) ratio of concentrated RTBO and MO, respectively. The response variables measured included concentrations of EPA, DHA, and EPA + DHA.

In Table 1, the experimental Factorial 2^2 design is shown. A total of 7 experiments were conducted, including 3 central point experiments to estimate experimental error. These experiments were performed in random order to minimize variability in the observed responses. The ALω-3s obtained were stored at −80 °C in amber bottles, with nitrogen to prevent oxidation.

### 2.6. Optimization of Enzymatic Acidolysis Process Variables Using Supercritical CO_2_ to Obtain ALω-3

Using response surface methodology (RSM) [27,28,29], the enzymatic acidolysis process was optimized by assessing the effects of supercritical CO_2_ temperature (°C) and pressure (bar) on the contents of EPA, DHA, and EPA + DHA. The following mathematical model was developed using RSM to predict the effect of the selected independent variables:

γ = β_0_ + ∑ β_i_X_i_ + ∑ β_ii_X_j_ + ∑∑β_ij_X_i_X_j_ + ε (1)

where β_0_, β_i_, β_ii_, and β_ij_ represent the regression coefficients for the intercept, linear, quadratic, and interaction terms, respectively, while X_i_, X_j_, and X_i_X_j_ denote the independent variables, and ε is the error term, accounting for variability not explained by the model. The estimation of regression coefficients was carried out using multiple regression analysis, applying a significance criterion of *p* < 0.05. Additionally, analysis of variance (ANOVA) was employed to evaluate the significance of both the individual regression terms and the overall model fit, also using a threshold of *p* < 0.05. All statistical computations were performed with Statgraphics Centurion XVI-2011 software 16.1.18 (StatPoint Technologies, Inc., Rockville, VA, USA).

A multiple response optimization analysis was performed to identify the optimal combination of experimental factor levels that simultaneously maximizes all response variables within the experimental design. This approach allowed for the simultaneous optimization of several dependent variables by determining the most favorable conditions of the independent factors.

The desirability function, ranging from 0 to 1, was employed to assess the degree to which a specific combination of factors satisfies the established optimization criteria. A desirability value of 0 represents an unacceptable outcome, whereas a value of 1 reflects an ideal response [10,21,23,30]. Through the integration of all response variables, a composite desirability score was calculated, enabling the prediction of optimal levels for EPA, DHA, and EPA + DHA.

### 2.7. Validation of the Optimal Antioxidant Lipid Formulation

Once the optimal ALω-3 formulation was obtained via enzymatic acidolysis under the predicted supercritical CO_2_ temperature and pressure conditions, experimental validation was performed. The formulation corresponding to the optimal point determined by the experimental design was evaluated under these conditions to confirm its accuracy. Thus, the optimal ALω-3 was validated and characterized by evaluating its EPA, DHA and EPA + DHA content using gas–liquid chromatography (GLC).

### 2.8. Identification and Quantification of Fatty Acids Using Gas Liquid Chromatography (GLC)

The fatty acid (FA) composition of ALω-3, Mω-3, and CMω-3 was determined by converting the samples into fatty acid methyl esters (FAMEs) through sequential alkaline and acid methylation, following the protocol established by IUPAC [31]. FA identification and quantification were carried out according to the AOCS Ce 1j-7 method [32], using a Shimadzu gas chromatograph (Kyoto, Japan) equipped with a flame ionization detector, split injection system, and an SPTM-2560 capillary column (100 m × 0.25 mm i.d. × 0.2 µm; Supelco, Bellefonte, PA, USA). The oven temperature was initially set at 160 °C for 3 min, followed by a gradual increase of 1 °C·min^−1^ up to 230 °C. Both the injector and detector were maintained at 240 °C, with hydrogen as the carrier gas. FA peaks were identified by comparing the retention times of the sample with those of a known standard. FAME identification was based on the reference standard GLC-463 (Nu-Chek Prep, Elysian, MN, USA), while the quantification of individual FAs (g·100 g^−1^ of total fatty acids, TFA) was performed using methyl tricosanoate (23:0 methyl ester) as an internal standard, in accordance with the AOCS method [32].

### 2.9. Identification and Quantification of Tocopherols and Tocotrienols

Tocopherol and tocotrienol contents were determined using high-performance liquid chromatography (HPLC), following the AOCS official method [33]. The HPLC system comprised a Merck-Hitachi L-6200A pump (Merck, Darmstadt, Germany), a Rheodyne 7725i injector equipped with a 20 μL sample loop, and a LiChro-CART Superspher Si 60 column (25 cm × 4 mm i.d., 5 μm particle size; Merck, Darmstadt, Germany). Detection was performed using a Hitachi Chromaster 5440 fluorescence detector, and chromatographic data were acquired and processed using Clarity chromatography software 2.4.1.43. on a PC. HPLC-grade hexane was added to 100 mg of the sample to a final volume of 10 mL in a volumetric flask. To quantify tocopherols and tocotrienols by HPLC, 80 µL of a standard solution containing 3 µg·mL^−1^ of tocopherols and tocotrienols was injected, and the corresponding peak areas were measured. Identification and quantification were carried out using the chromatograms obtained after 80 µL injections of the sample and its duplicate. The mobile phase was propan-2-ol in hexane (0.5/99.5 *v*/*v*) at a flow rate of 1 mL·min^−1^. Detection of the chromatographic peaks was performed at excitation and emission wavelengths of 290 nm and 330 nm, respectively. Tocopherols and tocotrienols from Calbiochem (Merck, Germany) were used as external standards. The results were reported as milligrams of tocopherols or tocotrienols per kilogram of oil, calculated according to the following equation:

(2) $$Tocopherols\;or\;Tocotrienols\;concentration=C*a*V*(A*m{)}^{-1}\;(mg\cdot kg^{-1}\;oil)$$

*C*: Standard concentration (µg mL^−1^);

*A*: Standard area (mVs);

*a*: Sample area (mVs);

*m*: Sample mass (g);

*V*: Volumetric flask volume (mL).

### 2.10. Thin-Layer Chromatography (TLC)

To identify the lipid species generated through the acidolysis reaction, a 1 μL aliquot of the antioxidant lipid samples was extracted and applied to a silica gel 60 F254 thin-layer chromatography (TLC) plate, along with the corresponding control samples. These included CMω-3, a mixture of concentrated rainbow trout belly oil rich in LCPUFAn-3, and MO (70/30, *w*/*w*) before acidolysis (Mω-3), along with a standard of tocopherols, gallic acid, and glycerol. For elution, a mixture of chloroform/acetone/acetic acid (96:4:1, *v*/*v*/*v*) was used, as described by Sabally et al. [34]. After completing the elution, the plate was stained with iodine vapor, and images of the results were captured for analysis. The order of elution on the chromatographic plate, from bottom to top, was determined according to their decreasing polarity as follows: monoacylglycerides (MAGs), free fatty acids (FFAs), diacylglycerides (DAGs), and triacylglycerides (TAGs) [34,35,36].

### 2.11. Differential Scanning Calorimetry (DSC)

Melting thermograms of long-chain polyunsaturated omega-3 fatty acids (LCPUFAn-3) from rainbow trout belly oil (RTBO) concentrate, the cold-pressed maqui seed oil (MSO), their mixture, and antioxidant lipids synthetized by enzymatic acidolysis under supercritical CO_2_ conditions were obtained using DSC. Melting profiles were determined using a PerkinElmer differential scanning calorimeter (PE DSC 7, Norwalk, CT, USA). Approximately 9–10 mg of each sample was placed in hermetically sealed aluminum pans. The thermograms were analyzed with the Pyris Player Software 11.0.0.0449 computer program, version 11.0.0.0449, where the temperatures (°C) of the beginning of the melting curve (TOnset), the maximum of the peaks (TPeak), and the end of the melting curve (TEndset) were obtained. Furthermore, the enthalpy of fusion (ΔH) was obtained from the TOnset and TEndset of the sample (J·g^−1^) [37,38]. The samples were initially rapidly heated (5 °C/min) from room temperature to 60 °C and held at this temperature for 10 min to destroy crystal memory. They were then cooled to −80 °C at 10 °C/min and held for 30 min, followed by a final heating to 60 °C at 5 °C/min to determine the melting profile.

### 2.12. Feeding Trial

Twenty-four male C57BL/6J mice (3 weeks) were obtained from the Public Health Institute of Chile. The animals were kept in the Animal Maintenance Unit of the Nutrition Department of the Faculty of Medicine, University of Chile, in a room maintained at a constant temperature of 21–23 °C, light and dark cycles of 12 h each, and were given access to their specific diet and water ad libitum. Following 2 weeks of acclimatization, the mice were fed with a high-fat diet (HFD) (60% fat, 20% protein 20% carbohydrates, D12492, Research Diets, New Brunswick, NJ, USA) for 8 weeks. Subsequently, the mice were switched to a control diet (CD) (10% fat, 20% protein, 70% carbohydrates, D12450B, Research Diets, New Brunswick, NJ, USA) and randomly divided into four groups with equal numbers of animals (*n* = 6), differentiated by the type of supplementation for 3 weeks, as follows: Glycerol (G; ACS grade, ≥99.5%, Sigma-Aldrich, Burlington, MA, USA)) (200 μL per day); commercial marine omega 3 (CMω-3; 400 mg EPA; 200 mg DHA per capsule) (75 μL per day; 32.4 mg EPA and 15.3 mg DHA); a mixture of concentrated RTBO, rich in LCPUFAn-3, and MO (70/30, *w*/*w*) (200 μL per day; 22.6 mg EPA and 36.1 mg DHA); and antioxidant lipids (ALω-3) (200 μL per day; 17.8 mg EPA and 26.9 mg DHA). All supplements were administered by oral gavage in fixed daily volumes, ensuring full intake. One week before euthanasia, an intraperitoneal glucose tolerance test (iGTT) was performed. After a 4 h fast at the end of the 12th week, the mice were weighed and then euthanized. The liver, visceral fat (fat dominated by abdominal cavity), and epididymal fat (visceral fat associated with metabolic dysfunction and whole-body insulin resistance [39,40]) were subsequently extracted, weighed using an analytical scale (Radwag AS60/220.r2), and stored at −80 °C until further analysis. All the procedures performed in this study were approved by the Institutional Animal Care and Use Committee (CICUA) of the University of Chile (Protocol n° 22567—MED—UCH).

### 2.13. Measurements of Biochemical Serum Parameters

Following blood collection via cardiac puncture, serum was obtained by centrifuging the samples at 3000× *g* for 15 min at room temperature. The activities of glutamate pyruvate transaminase (GPT), glutamate oxaloacetate transaminase (GOT), triacylglycerides (TG), total cholesterol (T-Chol), and HDL cholesterol (HDL-Chol) were measured using dry chemistry technology (SPOTCHEM EZ, Minneapolis, MN, USA). The serum insulin levels were quantified using an ultrasensitive mouse immunoassay kit (Mercodia, Uppsala, Sweden). Insulin resistance was estimated by calculating the homeostasis model assessment of insulin resistance (HOMA-IR) using the following formula:

HOMA-IR = [fasting glucose (mg/dL) × fasting insulin (µU/mL)]/405(3)

### 2.14. Histological Assessment

Liver samples were processed by dehydration, bleaching, and paraffin embedding for histological examination. Sections of 5 µm thickness were stained with Mayer’s hematoxylin and 1% aqueous eosin, which stained the nuclei blue and the cytoplasm pink. The histological slides were observed under a bright-field microscope (Leica DM500, Wetzlar, Germany), and 20–25 images were captured per sample. These images were analyzed using ImageJ software 1.54p (NIH, Bethesda, MD, USA) to quantify the percentage of steatosis. The images were converted to an 8-bit format, and threshold levels were adjusted to isolate the steatotic regions. The steatosis area was then quantified by measuring the area fraction in images taken at 400× magnification [20].

The steatosis score was determined according to the murine model described by Liang et al. [41], which incorporated assessments of macrovesicular steatosis, microvesicular steatosis, hypertrophy, and inflammation, with a total possible score of up to 12. The severity of both macrovesicular and microvesicular steatosis was independently evaluated and classified according to the percentage of the total affected area into the following four categories: 0 (<5%), 1 (5–33%), 2 (34–66%), and 3 (>66%). The distinction between macrovesicular and microvesicular steatosis was based on whether the lipid vacuoles displaced the nucleus to the periphery (macrovesicular) or not (microvesicular) [41]. The severity of hypertrophy and inflammation was determined similarly, as described by Liang et al. [41].

### 2.15. Quantitative PCR

Total RNA was extracted from the liver and visceral adipose tissue by homogenizing with Trizol reagent according to the manufacturer’s protocol. The remaining DNA was extracted with a DNA-free^TM^ DNA extraction kit from Invitrogen^TM^. Reverse transcription was then carried out to obtain 1 µg of cDNA using the Applied Biosystems^TM^ kit. Real-time PCR was performed using Brillant II SYBR Green qPCR master mix ST 600828 from Agilent Technologies. Primers used are listed in Table 2.

PCR amplification of the housekeeping gene β-actin was performed as a control. Ct values were determined using the AriaMx version 1.0.1406.2731 (Agilent Technologies, Santa Clara, CA, USA) when fluorescence was 25% greater than the background. PCR products were verified by melting-curve analysis. Relative expression levels were represented by the 2^−ΔΔCt^ method, corrected with the efficiency of each primer [42].

### 2.16. Total Antioxidant Capacity Assay Based on ABTS Oxidation

The total antioxidant capacity (TAC) was determined using a 96-well microplate assay based on the oxidation of 2,2′-azino-bis(3-ethylbenzothiazoline-6-sulfonic acid) (ABTS) induced by myoglobin and hydrogen peroxide (H_2_O_2_). The principle of this assay relies on the reaction between myoglobin and H_2_O_2_, which forms ferrylmyoglobin. This compound can oxidize ABTS to produce the ABTS cation radical (ABTS˙^+^), measurable at 600 nm. The oxidation of ABTS by myoglobin in the presence of H_2_O_2_ is expressed as a change in absorbance over the reaction time and converted to Trolox equivalents (TE) using a standard calibration curve. For the assay, 90 μL of PBS buffer (67 mM, pH 7.2), 50 μL of myoglobin solution (45 μM), 20 μL of ABTS solution (3 mM, final concentration: 300 μM), and 20 μL of diluted plasma (1:10) were mixed in each well. The mixture was incubated at 25 °C for 3 min. The reaction was initiated by adding 20 μL of H_2_O_2_, and the absorbance at 620 nm was recorded using a microplate reader (model: SUNRISE, Elisa-TECAN, Grödig, Austria) for 5 min at 25 °C. The change in absorbance (ΔAbs) was calculated using the absorbance values recorded 3 min after the reaction started, accounting for a 10 s delay in loading H_2_O_2_ into the wells. A calibration curve was constructed using Trolox as the standard (0–500 μM), and the results were expressed as micromoles of Trolox equivalents per liter (μM TE).

### 2.17. Statistics

Data are expressed as the mean ± standard error of the mean (SEM). Normality was assessed using the Shapiro–Wilk and Kolmogorov–Smirnov tests, and homoscedasticity was evaluated with Spearman’s rank correlation test to confirm the assumptions required for ANOVA. Statistical comparisons between and within groups were conducted using either one-way ANOVA followed by Tukey’s post hoc test, or the Kruskal–Wallis test followed by Dunn’s post hoc test, depending on data distribution. A *p*-value < 0.05 (95% CI) was considered statistically significant. All analyses were performed using GraphPad Prism 10.3.0 (San Diego, CA, USA).

## 3. Results

### 3.1. Enzymatic Acidolysis Under Supercritical CO_2_ of RTBO Concentrate and MO Using the Experimental Design by RSM

Enzymatic acidolysis under supercritical CO_2_ was carried out using a Factorial 2^2 experimental design with RSM. A total of seven enzymatically acidolysis experiments were conducted. Table 3 shows the effects of the independent variables—supercritical CO_2_ temperature and pressure—on the response variables, including EPA, DHA, and EPA + DHA contents.

**Figure 2:**
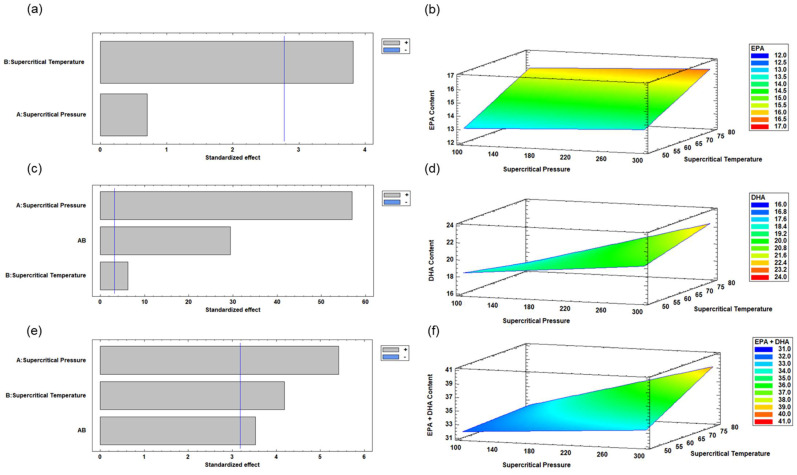
Effects of independent variables in the acidolysis process on EPA, DHA, and EPA + DHA (g/100 g TFA) contents. (**a**,**c**,**e**) Standardized Pareto diagram for the contents of the response variables EPA, DHA and EPA + DHA (g/100 g TFA), respectively. A higher value than the blue line mark indicates a significant effect (*p* < 0.05). (**b**,**d**,**f**) Estimated response surface diagram for the contents of the response variables EPA, DHA, and EPA + DHA (g/100 g TFA), respectively. Independent variables correspond to supercritical CO_2_ pressure (bar) and temperature (°C).

Figure 2 illustrates the effect of the independent variables in the acidolysis process on the EPA, DHA, and EPA + DHA contents. The standardized Pareto diagrams and estimated response surfaces for the contents of the response variables are depicted.

### 3.2. Optimization of Enzymatic Acidolysis Process Variables for RTBO Concentrate and MO Using Supercritical CO_2_ to Obtain an ALω-3

Table 4 (Part a) presents the optimization of the enzymatic acidolysis process variables, including supercritical CO_2_ temperature (°C) and pressure (bar), taking into account the information obtained regarding the contents of the response variables—EPA, DHA and EPA + DHA (Table 3).

### 3.3. Multiple Response Optimization of Enzymatic Acidolysis and Desirability

**Figure 3:**
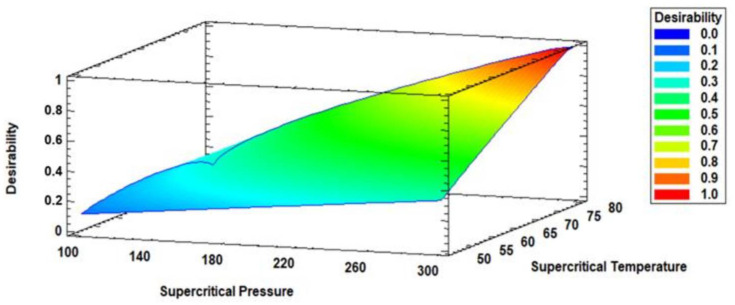
Response surface methodology (RSM) graphs for desirability value based on supercritical CO_2_ pressure (bar) vs. supercritical CO_2_ temperature (°C).

Table 4 (Part b) shows the results of the multiple response optimization, identifying the combination of factor levels that simultaneously maximize all responses within the experimental design. The optimized values for EPA, DHA, and EPA + DHA were 16.43, 22.44, and 38.87 g·100 g^−1^ TFA, respectively. The optimal conditions to achieve these values were 80 °C and 300 bar. The desirability function value was 0.99, indicating that this combination of factors meets the desired criteria (Figure 3). A desirability value of 0 represents an undesirable response, while a value of 1 indicates a highly desirable response.

### 3.4. Experimental Validation of the Optimal ALω-3 Formulation

Table 4 (Part c) provides the experimental validation of the multiple response optimization for the response variables in Part b. ALω-3 was validated using the optimized independent variables, i.e., 80 °C (supercritical CO_2_ temperature) and 300 bar (supercritical CO_2_ pressure). The results of the corresponding response variables were as follows: EPA, DHA, and EPA + DHA contents (g·100 g^−1^ total FAs) of 10.74, 16.23, and 26.97, respectively.

The Appendix A (Table A1) shows the adjusted linear (Y_1_) and quadratic (Y_2_ and Y_3_) polynomial equations for the predicted models of the EPA (Y_1_) (Figure 2b), DHA (Y_2_) (Figure 2d), and EPA + DHA contents (Figure 2f). Table A1 shows the regression coefficients of the first-order (Y_1_, Y_2_ and Y_3_) and second-order (Y_2_ and Y_3_) polynomial models for the different response variables. The results of fitting a multiple regression model describe the effect of the different process variables on the response variables—EPA, DHA and EPA + DHA (g·100 g−1 total FAs)—obtained from the enzymatic acidolysis of RTBO concentrate and MO under supercritical CO_2_.

### 3.5. Analysis of Characterization and Fatty Acid Composition of ALω-3

The fatty acid composition results obtained by gas-liquid chromatography (GLC) for the commercial marine Omega 3 (CMω-3), the mixture of LCPUFAn-3 concentrate and cold-pressed maqui seed oil (Mω-3), and the antioxidant lipids (ALω-3) are presented in Table 5, expressed as g fatty acids (FA)·100 g^−1^ total fatty acids (TFA) according to AOCS Ce 1j-07 [32]. A total of 20 fatty acids were identified, with the ALω-3 profile presenting significant differences with Mω-3 and CMω-3 (*p* < 0.05). The most prominent fatty acids in ALω-3 were linoleic (C18:2n-6), oleic (C18:1n-9), α-linolenic (C18:3n-3), eicosapentaenoic (EPA) (C20:5n-3), and docosahexaenoic (DHA) (C22:6n-3), with concentrations of 33.86 ± 0.09, 13.36 ± 0.07, 6.34 ± 0.01, 10.74 ± 0.13, and 16.23 ± 0.01 g FA·100 g^−1^ TFA, respectively. ALω-3 exhibited the highest palmitic acid (C16:0) content among the supplements, with 4.16 ± 0.01 g FA·100 g^−1^ TFA, significantly higher than Mω-3 (2.92 ± 0.08) and CMω-3 (0.53 ± 0.02) (*p* < 0.05). The fatty acid profile of ALω-3 showed 5.6% saturated fatty acids, 20.9% monounsaturated fatty acids, and 73.5% polyunsaturated fatty acids. Additionally, a 1:1 ratio was observed between the omega-6 and omega-3 fatty acids. It is worth mentioning that, following the enzymatic acidolysis process with lipase B from *Candida antarctica*, ALω-3 incorporated the fatty acids from both the cold-pressed maqui seed oil and the EPA and DHA from the LCPUFAn-3-concentrate of rainbow trout belly oil. CMω-3 exhibited approximately double the levels of EPA and DHA compared to other supplements, with EPA concentrations being four times higher. However, CMω-3 showed lower levels of linoleic and oleic acids.

Table 6 presents the concentrations of tocopherols and tocotrienols in ALω-3 determined through high-performance liquid chromatography–ultraviolet (HPLC-UV) analysis, as mg/kg of oil. The identified compounds included α, β, γ, and δ-tocopherols, as well as β and γ-tocotrienols. Only α-tocopherol was detected in trace amounts.

**Figure 4:**
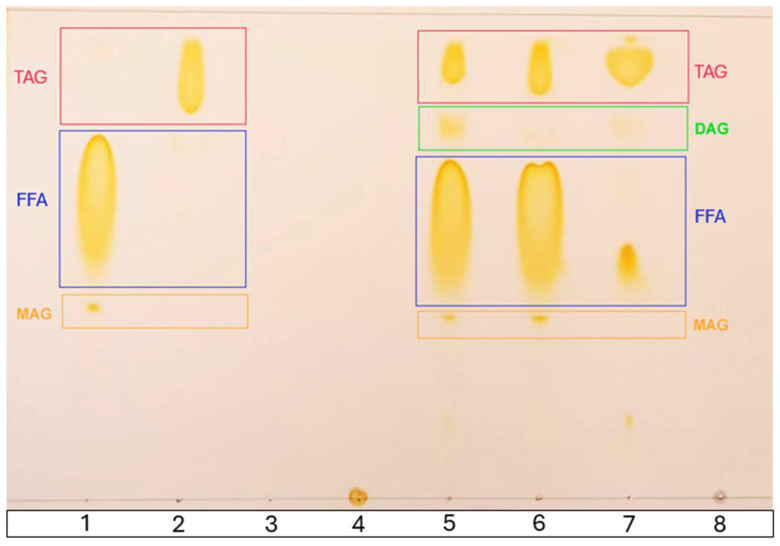
Thin-layer chromatography (TLC) reagents involved in antioxidant lipid (ALω-3) synthesis and murine supplementation. 1—LCPUFAn-3 concentrate, 2—cold-pressed maqui seed oil (MO), 3—tocopherol and tocotrienol standards, 4—gallic acid, 5—ALω-3 synthetized by enzymatic acidolysis of EPA/DHA concentrate and cold-pressed maqui seed oil under supercritical CO_2_ conditions, 6—mixture of LCPUFAn-3 concentrate plus cold pressed maqui seed oil (Mω-3), 7—Commercial marine Omega-3 (CMω-3), 8—glycerol (G). Mobile Phase: chloroform/acetone/acetic acid (96:4:1, *v*/*v*/*v*).

Lipid species were identified by thin-layer chromatography (TLC) for all reagents involved in both ALω-3 synthesis and murine supplementation (Figure 4). The rainbow trout belly oil (RTBO) concentrate, rich in LCPUFAn-3 EPA/DHA, consisted primarily of free fatty acids (FFAs) with low amounts of monoacylglycerols (MAGs) (Lane 1), while cold-pressed maqui seed oil (MO) was composed of triacylglycerols (TAGs) (Lane 2). The tocopherol and tocotrienol standards (Lane 3), gallic acid (Lane 4), and glycerol (Lane 8) did not elute with the mobile phase used. The elution profiles of ALω-3 (Lane 5) and Mω-3 (Lane 6) combined features from both substrates (LCPUFAn-3 concentrate and MO). ALω-3 displayed a more prominent region of diacylglycerols (DAGs)—products of acidolysis—confirming the enzymatic acidolysis process and structural modification achieved in this supplement. Finally, CMω-3 was primarily composed of TAG, along with a concentrated presence of FFA (Lane 8).

**Figure 5:**
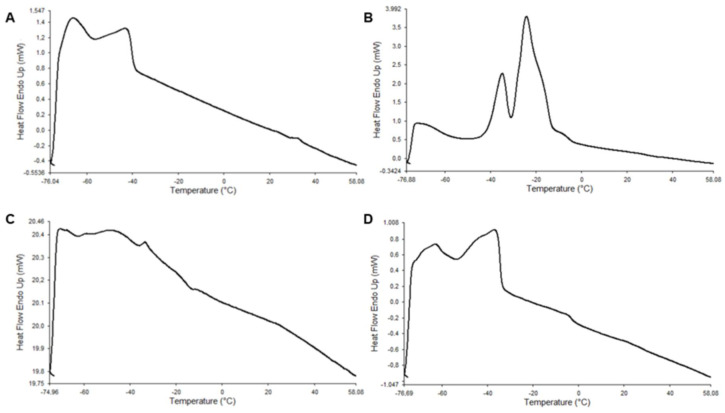
Melting thermogram of long-chain polyunsaturated omega-3 fatty acids (LCPUFAn-3) from rainbow trout belly oil (RTBO) concentrate (**A**), Cold-pressed maqui seed oil (MSO) (**B**), 70/30 mixture of LCPUFAn-3 RTBO concentrate and MSO (Mω-3) (**C**), and ALω-3, synthetized by enzymatic acidolysis of Mω-3 under supercritical CO_2_ conditions (**D**).

The melting thermograms of the reagents involved in the synthesis of ALω-3 are shown in Figure 5. The LCPUFAn-3 concentrate obtained from rainbow trout belly oil (Figure 5A) exhibited two predominant low-melting-point peaks, which were consistent with the melting behaviors of EPA and DHA, respectively. In contrast, the thermogram of MSO (Figure 5B) presented a broader thermal profile, with one low-melting-point peak at around –71.1 °C and two additional peaks at about –34.4 °C and –24.1 °C, reflecting a more complex lipid composition likely due to the presence of saturated and monounsaturated fatty acids.

The thermogram of the physical mixture Mω-3 (Figure 5C) displayed thermal transitions from both individual oils, with three peaks. This suggests a partial overlap and coexistence of lipid species from each source. In contrast, the ALω-3 thermogram (Figure 5D) exhibited a more defined and reorganized profile, with two main peaks at around –65.5 °C and –36.4 °C. The attenuation of the lower-intensity peak and the shift of melting transitions suggest structural modifications in the triacylglycerol backbone due to enzymatic acidolysis and the possible formation of new lipid species. These thermal transitions are summarized in Table 7, including ΔH and **the** onset (TOnset), endset (TEndset), and peak (TPeak) temperatures. Overall, the data provide a distinct thermal fingerprint of the individual reagents, their physical mixture, and the final lipid ALω-3, supporting the occurrence of lipid restructuring during synthesis.

### 3.6. ALω-3 Effects on Tissue Weight of HFD Murine Model

**Figure 6:**
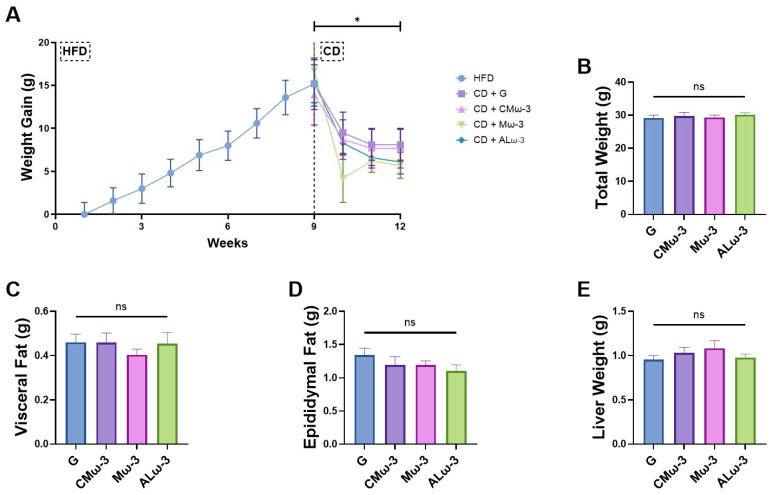
Total weight, visceral fat weight, epididymal fat weight, and liver weight of high-fat diet (HFD) murine model after the different reversion treatments. Monitoring weight gain and loss of mice (**A**); total weight (**B**); visceral fat weight (**C**); epididymal fat weight (**D**); and liver weight (**E**) at the end of the treatment. Male C57BL/6 mice were fed a HFD for 8 weeks and then switched to a control diet (CD), along with supplementation for 3 weeks. Supplementation corresponded to glycerol (G), commercial marine omega-3 (CMω-3), a mixture of EPA/DHA concentrate and cold-pressed maqui seed oil (Mω-3), or the antioxidant lipids (ALω-3) synthetized by enzymatic acidolysis of Mω-3 under supercritical CO_2_ conditions. Data are expressed as mean ± standard error of the mean (S.E.M.). The mice were fasted for 6 h prior to euthanasia (*n* = 6 mice for each group). Statistical differences were determined by one-way ANOVA, followed by Tukey’s post hoc test: * *p* < 0.05 and ns = not significant.

Obesity is characterized by excessive fat gain and increased distribution toward visceral fat storage, resulting in an increase in the individual’s total weight. Habit changes, including dietary modification and physical activity, are the most effective in managing this disease. In this study, the weight of the mice was monitored during the various treatments until the sacrifice of the mice (Figure 6). The mice were fed a high-fat diet (HFD) for 8 weeks (acclimatation week prior). Then, the mice were randomly divided into four groups, switching to a control diet (CD) supplemented with ALω-3, Mω-3, CMω-3, or G for 3 weeks (Figure 6A). During the HFD phase, the mice reached a maximum weight of approximately 40 g. Upon switching to the CD plus supplementation, there was a significant reduction in their total weight, compared to the weight prior to supplementation (Figure 6A). Additionally, at the end of the treatment, the total body weight, along with the weights of the visceral and epididymal fats, and the liver, were measured to assess the effects of the different supplementation regimens on each group (Figure 6B–E). None of these parameters showed statistical differences among the four treatment groups. Overall, all treatments resulted in an average total body weight of 29.62 ± 0.43 g, with visceral and epididymal fat percentages (calculated as g fat/g total weight × 100) of 1.49% and 4.06%, respectively, and a liver weight of 1.01 ± 0.00 g. None of the treatments contributed to reducing body weight nor the proportion of visceral fat. Although the supplementation with ALω-3 presented the biggest reduction in epididymal fat compared to the G group (i.e., equivalent to a decrease of 17.7%) and the groups supplemented with Mω-3 (i.e., equivalent to a decrease of 11.0%) and CMω-3 (i.e., equivalent to a decrease of 10.7%), this reduction was not statistically significant (*p* > 0.05).

### 3.7. ALω-3 Effects on Biochemical Parameters of HFD Murine Model

**Figure 7:**
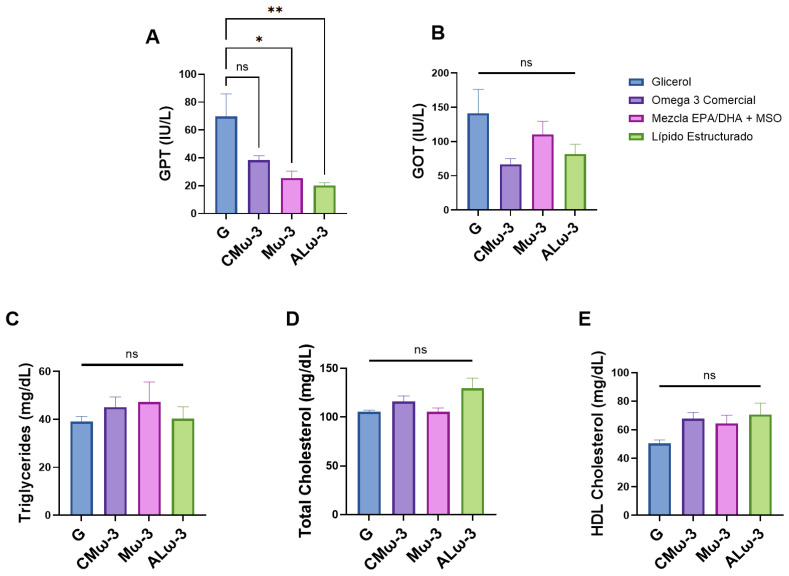
Serum parameters of high-fat diet (HFD) murine model after the different reversion treatments associated with MASLD and dyslipidemia. Serum glutamate pyruvic transaminase (GPT) (**A**), serum glutamate oxaloacetic transaminase (GOT) (**B**), triacylglycerols (**C**), total cholesterol (**D**), and high-density lipoprotein (HDL) cholesterol (**E**). Male C57BL/6 mice were fed a HFD for 8 weeks and then switched to a control diet (CD) with supplementation for 3 weeks. Supplementation corresponded to glycerol (G), commercial marine Omega-3 (CMω-3), a mixture of EPA/DHA concentrate and cold-pressed maqui seed oil (Mω-3), or antioxidant lipids (ALω-3) synthetized by enzymatic acidolysis of mixture under supercritical CO_2_ conditions. The mice were fasted for 6 h prior to euthanasia (*n* = 6 mice for each group). Statistical differences were determined using the Kruskal–Wallis test, followed by Dunn’s comparative test: * *p* < 0.05; ** *p* < 0.01 and ns = not significant.

The biochemical parameters were analyzed to assess the potential of ALω-3 supplementation in mitigating liver damage associated with MASLD and dyslipidemia (Figure 7). Compared to the G group, ALω-3 supplementation resulted in a significantly greater reduction in serum glutamate pyruvate transaminase (GPT) levels (i.e., equivalent to a decrease of 71.0%; *p* < 0.01); Mω-3 supplementation resulted in a significant reduction in serum GPT levels (i.e., equivalent to a decrease of 63.6%; *p* < 0.05); and CMω-3 supplementation showed a trend toward reducing GPT levels, but this reduction was not significant (i.e., equivalent to a decrease of 45.0%; (*p* > 0.05), (Figure 7A).

Regarding glutamate oxaloacetate transaminase (GOT) levels, all supplementations exhibited a trend toward reduction compared to G. The greatest decrease was observed with ALω-3, showing a 42% reduction, while Mω-3 showed a 21.8% decrease compared to G. However, these changes were not statistically significant (*p* > 0.05) (Figure 7B).

No significant differences in triacylglycerols (TAGs) (Figure 7C) were observed with supplementation (*p* > 0.05). ALω-3 supplementation displayed similar TAG values to the G group, without the increases observed in the Mω-3 and CMω-3 groups. Notably, despite the higher caloric content of ALω-3 supplementation, its behavior resembled that of the glycerol-supplemented group. Although none of the supplementations resulted in significant differences in total cholesterol (T-Chol) (Figure 7D) or high-density lipoprotein cholesterol (HDL-Chol) *(p* > 0.05), T-Chol and HDL-Chol increased with ALω-3 supplementation (Figure 7E).

### 3.8. Effect of ALω-3 on MASLD-Associated Liver Damage

**Figure 8:**
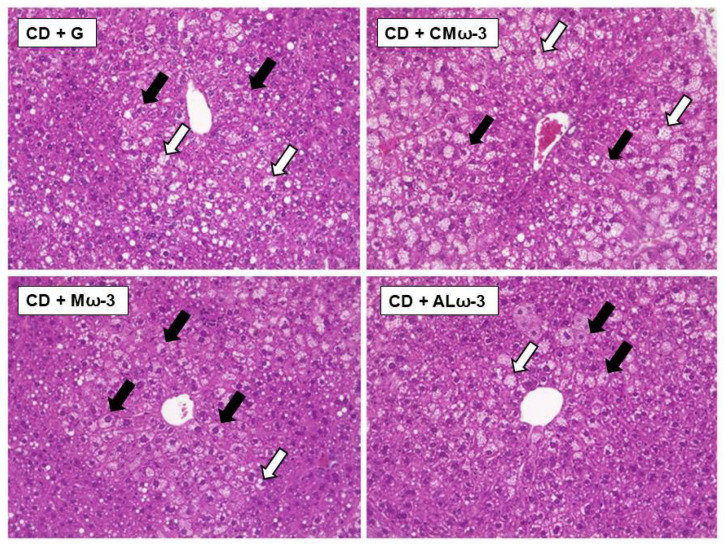
Histology of liver steatosis of high-fat diet (HFD) murine model after the different reversion treatments associated with MASLD. Hematoxylin and Eosin (H&E) staining of paraffin liver sections displaying general architecture and cellular morphology. White arrows indicate macrovesicular steatosis and black arrows microvesicular steatosis. Male C57BL/6 mice were fed a HFD for 8 weeks and then switched to a control diet (CD) with supplementation for 3 weeks. Supplementation corresponded to glycerol (G), commercial marine omega-3 (CMω-3), a mixture of EPA/DHA concentrate and cold-pressed maqui seed oil (Mω-3), or antioxidant lipids (ALω-3) synthetized by enzymatic acidolysis of mixture under supercritical CO_2_ conditions. *n* = 6 mice for each group. Magnification of the figure is 400×.

Considering the results in Section 3.7, a histological analysis of liver samples from mice in each group was performed using hematoxylin and eosin (H&E) staining (Figure 8). The steatosis score was calculated from two sections per animal across the different treatment groups (Table 8). To this end, a hepatic steatosis classification system for mice, developed by Liang et al. [41], was used to evaluate the severity of histopathological features based on their frequency within the tissue (Section 2.13). These features include microvesicular steatosis, macrovesicular steatosis, hypertrophy, and the number of inflammatory foci per field (Table 8). In the liver tissues of the ALω-3 group, microvesicular steatosis was predominantly observed, with smaller amounts of macrovesicular steatosis followed by hypertrophy and inflammatory foci (Figure 8). Using the murine scoring system, macrosteatosis and hepatocyte hypertrophy were scored as 0 across all groups, as less than 5% of the area was affected. Similarly, inflammatory foci per field were also scored as 0, given that the ratio remained below 0.5. Microvesicular steatosis was assigned a score of 1 in all groups, corresponding to an affected area of 5–33%, with specific percentages for groups G, CMω-3, Mω-3, and ALω-3 being 10.6%, 10.0%, 8.02%, and 8.03%, respectively. Consequently, supplementation did not show a significant effect in reducing the microvesicular steatosis score (Table 8), indicating a mild degree of steatosis and no inflammation across all groups.

### 3.9. Evaluation of Glucose Homeostasis After ALω-3 Supplementation

**Figure 9:**
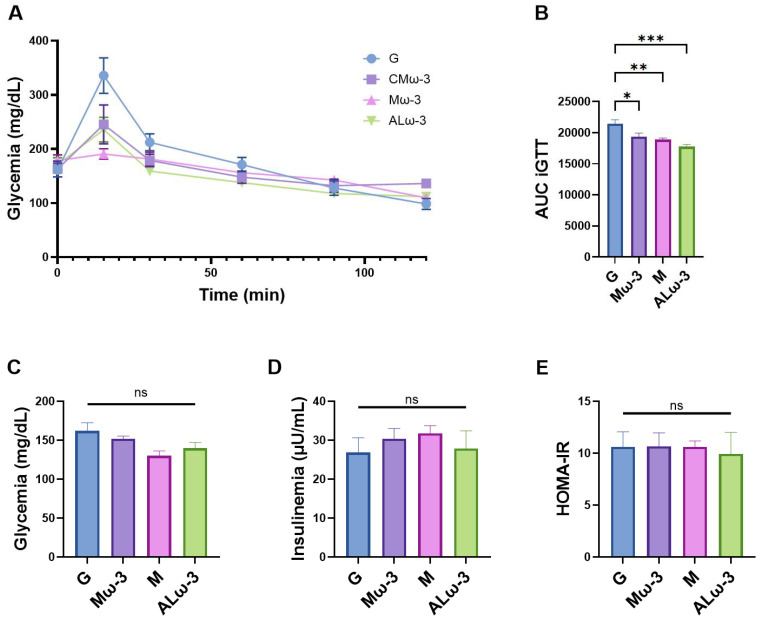
Glucose homeostasis parameters of high-fat diet (HFD) murine model after the different reversion treatments. Intraperitoneal glucose tolerance test (iGTT) (**A**) performed after 4 h of fasting by administration of 5 g/kg of glucose. Area under the curve (AUC) of A (**B**). Fasting blood glucose concentration (**C**), fasting serum insulin concentration (**D**) at the end of treatment. HOMA-IR (**E**) was calculated by: [fasting blood glucose (mg/dL) × fasting insulin (μU/mL)]/405. Male C57BL/6 mice were fed a HFD for 8 weeks and then switched to a control diet (CD) with supplementation for 3 weeks. Supplementation corresponded to glycerol (G), commercial marine omega-3 (CMω-3), a mixture of EPA/DHA concentrate and cold-pressed maqui seed oil (Mω-3), or the antioxidant lipids (ALω-3) synthetized by enzymatic acidolysis of the mixture under supercritical CO_2_ conditions. Data are expressed as mean ± standard error of the mean (S.E.M.). The mice were fasted for 6 h prior to euthanasia (*n* = 6 mice for each group). Statistical differences were determined by one-way ANOVA test, followed by Tukey’s post hoc test: * *p* < 0.05; ** *p* < 0.01; *** *p* < 0.001 and ns = not significant.

To assess glucose homeostasis, an intraperitoneal glucose tolerance test (iGTT) was conducted one week prior to euthanasia (Figure 9A,B). Additionally, fasting serum glucose and insulin levels were measured to calculate the homeostatic model assessment for insulin resistance (HOMA-IR) at the end of the treatment period (Figure 9C–E). Supplementation with CMω-3, Mω-3, and ALω-3 significantly reduced the area under the curve (AUC) of the iGTT compared to the G group, with reductions of 9.7% (*p* < 0.05), 12.0% (*p* < 0.01), and 17.3% (*p* < 0.001), respectively (Figure 5B); ALω-3 showed the greatest reduction and achieved the highest level of statistical significance. Notably, lower fasting serum glucose levels were observed in the groups supplemented with Mω-3 and ALω-3 compared to the G group (i.e., corresponding to decreases of 19.8% and 13.8% for Mω-3 and ALω-3, respectively); however, no significant differences were found among the four groups (*p* > 0.05) (Figure 9C). Regarding the fasting insulin levels, supplementation with CMω-3, Mω-3, and ALω-3 did not significantly reduce serum concentrations (Figure 9D). However, ALω-3 supplementation was notable for not causing an increase in the insulin levels as compared to CMω-3 and Mω-3 (*p* > 0.05). These results align with the observed variations in the HOMA-IR index (Figure 9E). Therefore, these results indicate that ALω-3 supplementation improves glucose tolerance without increasing insulin sensitivity.

### 3.10. Effect of Supplementation with ALω-3 on the Transcription of Pro-Inflammatory Genes

To evaluate whether ALω-3 supplementation alongside a normal-fat diet could mitigate hepatic pro-inflammatory markers, gene expression of the cytokines (IL-6 and TNFα) and TLR4 in mouse liver and visceral fat homogenates was analyzed (Table 9). The expression of these markers was compared with the G group and normalized to the expression of the housekeeping gene β-actin. None of the supplementations (CMω-3, Mω-3, and ALω-3) significantly reduced the expression of pro-inflammatory markers in liver and visceral fat, except for Mω-3 supplementation, which significantly decreased IL-6 expression in visceral fat (*p* < 0.05).

In liver samples, only ALω-3 supplementation reduced IL-6 expression (*p* > 0.05) by 6.7% with respect to G. Additionally, Mω-3 and ALω-3 supplementation showed a trend toward reduced expression (*p* > 0.05) of TNF-α, with reductions of 15.1% and 24.5% for both treatments with respect to G. Mω-3 and ALω-3 supplementation also showed reductions (*p* > 0.05) of 17.5 (Mω-3) and 31.2% (ALω-3) for TLR4 with respect to G supplementation.

In visceral fat, Mω-3 supplementation exhibited significantly reduced expression of IL-6 (*p* < 0.05), corresponding to a 61.8% reduction with respect to G. A reduction in IL-6 of 18.6% (*p* > 0.05) was observed with CMω-3 supplementation, with respect to G. Mω-3 supplementation increased TNF-α expression by 6.0% with respect to G, whereas CMω-3 and ALω-3 reduced TNF-α expression by 10% compared to G (*p* > 0.05). TLR4 decreased by 27.2% with CMω-3 supplementation and 38.5% with Mω-3 supplementation (*p* > 0.05), while ALω-3 supplementation did not display a similar pattern with respect to G.

### 3.11. Evaluation of Total Antioxidant Capacity (TAC) in Serum After ALω-3 Supplementation

Total antioxidant capacity (TAC) in serum serves as a comprehensive indicator of the antioxidant defenses present in the bloodstream, reflecting the ability to neutralize free radicals and mitigate oxidative stress. Evaluating serum TAC provides insights into the systemic effects of dietary interventions on oxidative balance, particularly in conditions such as obesity and MASLD, where oxidative stress is a key contributor to disease progression.

**Figure 10:**
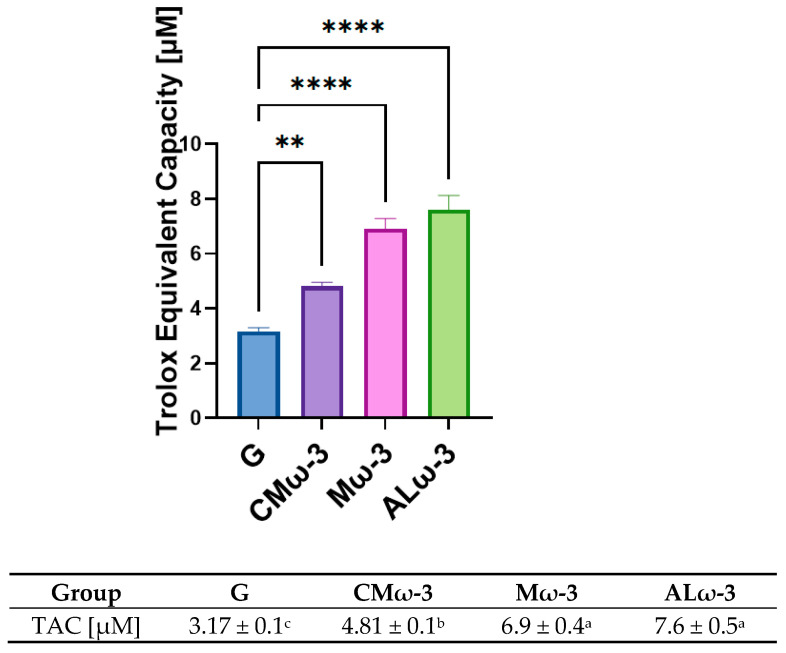
Serum antioxidant capacity (TAC) of high-fat diet (HFD) murine model after the different reversion treatments. TAC was assessed in serum to evaluate the antioxidant potential of the dietary interventions. Male C57BL/6 mice were fed a HFD for 8 weeks and then switched to a control diet (CD) with supplementation for 3 weeks. Supplementation corresponded to glycerol (G), commercial marine omega-3 (CMω-3), a mixture of EPA/DHA concentrate and cold-pressed maqui seed oil (Mω-3), or antioxidant lipids (ALω-3) synthetized by enzymatic acidolysis of the mixture under supercritical CO_2_ conditions. Data are presented as mean ± standard error of the mean (S.E.M.), with six mice per group (*n* = 6 mice for each group). Mice were fasted for 6 h prior to euthanasia. Statistical differences between groups were determined using one-way ANOVA followed by Tukey’s multiple comparison test: ** *p* < 0.01; **** *p* < 0.0001; ns = not significant. Values with different superscript letters (^a–c^) indicate significant differences (*p* < 0.05).

Figure 10 shows the TAC results in serum for the four post-HFD reversal treatments. The G group, which did not receive antioxidant supplementation, showed the lowest TAC value (3.17 ± 0.1 μM). The CMω-3 group, which were supplemented with commercial marine omega-3, showed a significant increase in TAC (4.81 ± 0.1 μM) compared to the G group (*p* < 0.01), but the value was not as high as those in the other groups. The Mω-3 group (supplemented with a mixture of EPA/DHA concentrate and cold-pressed maqui seed oil) showed a significant improvement in TAC (6.9 ± 0.4 μM), surpassing the CMω-3 group (*p* < 0.001) and showing an intermediate value between the supplemented groups. The ALω-3 group (supplemented with antioxidant lipids composed of EPA/DHA concentrated and cold-pressed maqui seed oil) showed the highest TAC value (7.6 ± 0.5 μM), significantly higher than all other groups, including CMω-3 and Mω-3 (*p* < 0.001).

## 4. Discussion

In the present study, ALω-3 antioxidant lipids were synthesized by enzymatic acidolysis with EPA/DHA concentrate and Maqui (*Aristotelia chilensis* (Mol.) Stuntz), characterized, and their intake evaluated in a C57BL/6 murine model fed a HFD, with a specific focus on the reversal of metabolic and inflammatory alterations associated with metabolic syndrome. The effects of switching from a HFD to a regular-fat diet (control diet) supplemented with ALω-3 were evaluated in mice, assessing total body weight, liver weight, visceral and epididymal fat, biochemical parameters (hepatic injury and dyslipidemia markers), glucose metabolism, systemic inflammation in both liver and visceral fat, and total antioxidant capacity in serum.

### 4.1. Optimization of Enzymatic Acidolysis Conditions Using Supercritical CO₂

This study employed a factorial 2^2 design combined with response surface methodology (RSM) to optimize the enzymatic acidolysis between an RTBO concentrate and MO under supercritical CO_2_ conditions. This approach enabled the identification of relevant trends and the development of a statistically significant predictive model *(p* < 0.05), making it appropriate for optimization studies [27]. Regarding the effects of the studied variables, both temperature and CO_2_ pressure significantly influenced the contents of EPA, DHA, and their mixture (EPA + DHA). It was observed that high pressures (300 bar) and elevated temperatures (80 °C) favored greater incorporation of these polyunsaturated fatty acids, reaching maximum predicted values of 16.43, 22.44, and 38.87 g/100 g TFA for EPA, DHA, and EPA + DHA, respectively, under optimized conditions. Similarly, previous studies have reported this behavior of temperature and supercritical pressure on EPA and DHA contents [21,23,30]. This behavior can be attributed to the enhanced solvent power of supercritical CO_2_ at high pressures, which improves substrate solubilization and biocatalytic efficiency by facilitating enzyme–substrate interactions [43,44,45]. These results confirm the potential of the supercritical CO_2_ enzymatic system for the synthesis of ω-3-rich lipids.

### 4.2. Characterization and Thermal Analysis of ALω-3

The fatty acid profile of ALω-3 revealed that oleic, linoleic, and α-linolenic acids were present at 13.4%, 33.9%, and 6.3%, respectively, primarily contributed by MO. These findings align with those reported by Sanchez et al. [10] in their analysis of freeze-dried maqui seed oil, where oleic, linoleic, and α-linolenic acids accounted for 33.5%, 50.5%, and 1.6%, respectively. In our study, the synthesis of ALω-3 used a 70/30 (*w*/*w*) mixture of the LCPUFAn-3 concentrate and MO, which explains the comparable profiles. Linoleic and α-linolenic acids are essential fatty acids, crucial for their nutritional importance, as they cannot be synthesized by the human body and must be obtained through the diet [46]. Additionally, ALω-3 contained omega-3 fatty acids, particularly EPA + DHA, at a concentration of 26.94 ± 0.14 g FA·100 g^−1^ TFA, derived from the LCPUFAn-3 concentrate. Similarly, Singh et al. [47] synthesized a lipid from perilla seed oil and palm olein via enzymatic interesterification using immobilized Lipozyme TL IM lipase. Their lipid contained 34.5% oleic acid, primarily from palm olein, and 12.6% linoleic acid and 16.2% α-linolenic acid, predominantly sourced from perilla seed oil [47]. Notably, ALω-3 showed a higher palmitic acid content than the other supplements, likely due to enzymatic acidolysis altering fatty acid distribution. Nevertheless, its lipid profile remained rich in polyunsaturated fatty acids, which are metabolically beneficial.

In terms of tocopherol and tocotrienol composition, ALω-3 contained β-tocopherol (22.9 ± 1.4 mg/kg oil), γ-tocopherol (6.8 ± 0.7 mg/kg oil), δ-tocopherol (22.4 ± 4.7 mg/kg oil), β-tocotrienol (24.9 ± 0.2 mg/kg oil), and γ-tocotrienol (22.9 ± 1.7 mg/kg oil); α-tocopherol was detected in trace amounts. In comparison, Sánchez et al. [10] reported that freeze-dried maqui seed oil contained 735 ± 19 mg/kg of α-tocopherol, 5 ± 1 mg/kg of β-tocopherol, 97 ± 4 mg/kg of γ-tocopherol, and 7 ± 1 mg/kg of δ-tocopherol. Additionally, Bastias-Montes et al. [48] analyzed cold-pressed maqui seed oil and found α-tocopherol (169.3 ± 11.4 mg/kg oil), β-tocopherol (7.8 ± 2.3 mg/kg oil), γ-tocopherol (56.8 ± 2.9 mg/kg oil), δ-tocopherol (13.6 ± 3.5 mg/kg oil), β-tocotrienol (20.2 ± 5.9 mg/kg oil), and γ-tocotrienol (53.9 ± 7.4 mg/kg oil).

The composition of tocopherols and tocotrienols in ALω-3 may be influenced by the enzymatic acidolysis process in SCCO_2_. The specific temperature and pressure conditions used in this process likely prioritize the acidolysis of omega-3 fatty acids and other compounds, potentially reducing the tocopherol and tocotrienol levels [49,50]. Notably, while no daily intake recommendations exist for tocotrienols, they possess unique properties not found in tocopherols [14]. The diversity of tocopherol and tocotrienol composition highlight the potential of ALω-3 as a functional lipid with distinct antioxidant properties, offering a possible alternative to more commonly used oils with only α-tocopherol content.

TLC was used to assess the lipid identity of the ALω-3 components, confirming the effectiveness of enzymatic acidolysis between the LCPUFAn-3 concentrate and MO due to the presence of secondary reaction products—mono- and diacylglycerols. Hamam and Shahidi [51] optimized the synthesis of structured lipids through the acidolysis of high-laurate canola oil with DHA or EPA and analyzed the positional distribution of these LCPUFAn-3 in the glycerol backbone. Similarly, Dobale-Rosaval et al. [52] aimed to synthesize structured acylglycerols (sAGs) using deodorized, refined commercial salmon oil as the substrate. Intra- and interesterification was catalyzed by an immobilized nonspecific lipase B from *Candida antarctica* under SCCO_2_ conditions [52]. This lipase exhibits non-specific catalytic activity, promoting the random esterification of fatty acids at the sn-1, sn-2, and sn-3 positions of the glycerol backbone. EPA and DHA are more efficiently absorbed when they are located at the sn-2 position, due to the easier formation of micelles [53].

Differential scanning calorimetry (DSC) is a well-established technique for evaluating the thermal behavior and physicochemical properties of lipid systems. In this study, the thermal profile of the lipid ALω-3 showed notable differences compared to the profiles of its precursor oils. Specifically, changes in the number, shape, and positional distribution of the melting peaks suggest molecular reorganization within the triacylglycerol matrix, which cannot be attributed to simple mixing of the components. These thermal transitions are associated with alterations in the crystalline morphology of the lipids, which reflect changes in chemical composition and fatty acid positioning. As previously reported, the melting behavior of lipids is strongly influenced by their structural configuration, including the distribution of saturated and unsaturated fatty acids at the sn-positions of the glycerol backbone [54]. Therefore, DSC provides indirect but meaningful evidence supporting the formation of new lipids by enzymatic acidolysis. Similar applications of DSC have been reported in the literature for characterizing lipid restructuring following enzymatic or physical modification processes [54,55,56]. While this method does not provide positional information on fatty acids, it complements chromatographic and compositional analyses by revealing thermal signatures associated with structural lipid changes.

### 4.3. Impact of ALω-3 Supplementation on Body Weight and Fat Distribution in a High-Fat Diet-Induced Obesity Murine Model

Following the characterization of ALω-3, its dietary supplementation was evaluated in an animal model. Male C57BL/6 mice were selected for the study and initially fed a HFD for 8 weeks. After this period, the mice were randomly assigned to four groups, transitioning from the HFD to a CD. Each group received one of the following supplementations: G, CMω-3, Mω-3, or ALω-3. Based on the monitoring of weight changes in mice, transitioning from an HFD to a CD significantly reduced total body weight, with a marked decrease observed after treatment (*p* < 0.001). The weights of visceral fat, epididymal fat, and liver were also measured at the end of the supplementation period. No significant differences were found among all the supplementation groups regarding the total body, liver, or fat weights (*p* > 0.05). Although ALω-3 supplementation resulted in the largest reduction in epididymal fat compared to the glycerol (17.7%) and other groups (11.0% for Mω-3 and 10.7% for CMω-3), the reductions were not statistically significant (*p* > 0.05). This suggests that the supplementation with ALω-3 may have a slight reduction on fat deposits, but its impact on body weight and fat distribution is not conclusive in this study. Notably, Ji et al. [57] suggested that while caloric intake reduction effectively decreases body weight, fat mass, and obesity-induced ectopic fat deposition in the liver, it is insufficient on its own to fully prevent or manage the metabolic disorders associated with this condition.

### 4.4. Effects of ALω-3 on Biochemical Parameters Related to MASLD and Lipid Profile in HFD-Fed Mice

GPT, GOT, and GGT are intracellular enzymes that catalyze transamination reactions and are present in various tissues throughout the body. Among these, GPT and GGT are the most specific markers for detecting liver damage, as their elevated levels in the bloodstream are closely associated with hepatic injury [58].

The results show that ALω-3 supplementation led to a significantly greater reduction in GPT levels compared to the G group. Specifically, ALω-3 supplementation resulted in a 71.0% decrease in GPT levels (*p* < 0.01), while Mω-3 supplementation resulted in a 63.6% reduction (*p* < 0.05). In contrast, CMω-3 supplementation showed a trend toward reducing GPT levels; however, the reduction was not statistically significant, with a decrease of 45.0% (*p* > 0.05). For GOT levels, all supplementation groups exhibited a trend toward reduction compared to the G group. However, the most notable reduction was observed in the ALω-3 group, with a decrease of 42%, while Mω-3 resulted in a 21.8% reduction, although none of these changes were statistically significant (*p* > 0.05). These findings suggest a potential hepatoprotective effect, which could be attributed to the protective effects of omega-3s in LCPUFAn-3, as well as those of tocopherol and tocotrienols from MO.

A previous study by Valenzuela et al. [59] investigated the supplementation of an encapsulated fish-oil-derived LCPUFA-n3 concentrate in the prevention of fatty liver disease alongside HFD-fed mice C57BL/6J over 12 weeks. LCPUFA-n3 supplementation resulted in a 21.4% and 4.2% reduction in GPT and GOT serum levels, respectively (compared to a HFD group), although these changes were not statistically significant (*p* > 0.05) [59]. On the other hand, a reversal study by Rodriguez-Echevarria et al. [60] evaluated the effects of switching diets or the intraperitoneal administration of either 18-HEPE or 17-HDHA in mice previously fed with HFD for 16 weeks. This study demonstrated that these fatty acids differentially exert hepatoprotective effects through the upregulation of nuclear receptors PPARα/γ and improvements in serum adipokine profiles [60]. Additionally, the study showed that a diet-switch regimen serves as a selective therapeutic approach, as most NAFLD markers (SREBP-1, PPARγ, and NF-κB) and histological alterations were significantly ameliorated by this intervention [60]. Moreover, Claría et al. [20], in a prevention study, evaluated the supplementation of cold-pressed maqui seed oil (MO) in HFD-fed C57BL/6 mice over 12 weeks. This intervention resulted in 52.4% and 28.7% reductions in serum GPT and GOT levels (*p* < 0.05), respectively, compared to HFD-fed mice supplemented with sunflower oil [20]. Finally, Valenzuela et al. [61] investigated the supplementation of a mixture of LCPUFA-n3 concentrate and extra virgin olive oil (EVOO) in mice fed an HFD for 12 weeks. This treatment reduced serum GPT and GOT levels by 4.8% and 3.9%, respectively, between the HFD-fed groups, with no significant differences observed between the supplementation of LCPUFA-n3 or EVOO individually [61]. Another study examining the impact of structured lipids on transaminase levels is Dovale-Rosabal et al. [30], where supplementation with structured phenolic acylglycerols containing gallic acid (sPAG) in HFD-fed mice did not lead to an increase in transaminase levels compared to a group supplemented with sunflower oil. Similarly, Martínez-Galán et al. [62] reported that feeding mice an HFD with 50% lipid content in the form of structured lipids (SLs) enriched with capric acid, derived from grape seed oil for 8 weeks resulted in a 2.4% and 3.7% reduction in GPT and GOT levels, respectively, compared to an HFD with 50% lipid content of grape seed oil.

Based on these results, further assessment of MASLD conditions in the mouse groups was conducted (Figure 4). Liver biopsy remains the primary tool for diagnosing and monitoring MASLD [41]. The histological analysis of ALω-3 group liver sections revealed mainly microvesicular steatosis and, to a lesser extent, macrovesicular steatosis, with a higher proportion in the G group. However, when using the murine steatosis scoring system by Liang et al. [41], no significant variations were observed (*p* > 0.05). Microvesicular steatosis may indicate improved lipid droplet metabolism, as seen in a study by La Fuente et al. [63], where exercise mitigated hepatic damage in a murine MASLD model by reducing lipid droplet size and normalizing protein markers of de novo lipogenesis and lipolysis.

The lipid profile, which includes measurements of triacylglycerols (TAGs), total cholesterol (T-Chol), and high-density lipoprotein cholesterol (HDL-Chol), is widely used to assess cardiovascular risk associated with lipid metabolism disorders. Elevated TG and T-Chol levels are well-established risk factors for cardiovascular disease, while HDL-Chol is considered protective [64]. The American Heart Association (AHA) recommends the prescription of n-3 fatty acids (EPA + DHA or EPA only) at doses of ≥3 g/day as a safe and effective approach for lowering TG levels, either alone or in combination with other lipid-lowering agents [65]. In this study, no significant differences were observed in the serum levels of TAG, total cholesterol (T-Chol), or high-density lipoprotein cholesterol (HDL-Chol) among the four groups of mice. ALω-3 supplementation displayed similar TG levels to the G group, showing no increase in TG levels unlike the Mω-3 and CMω-3 groups. Interestingly, despite the higher caloric content of the ALω-3 supplementation, it exhibited a behavior similar to the glycerol-supplemented group, suggesting that ALω-3 may not contribute to the TG increases seen in the Mω-3 and CMω-3 groups. Furthermore, according to Quimby & Luong [66], none of the groups showed abnormal values for these parameters, suggesting a positive effect of the reversion intervention itself. The lack of reduction in the serum lipid levels of the ALω-3-supplemented mice aligns with the findings from Dovale-Rosabal et al. [30], where supplementation with structured phenolic acylglycerols (sPAGs) in HFD-fed mice failed to reduce TG and T-Chol levels.

### 4.5. Effect of ALω-3 Supplementation on Glucose Homeostasis and Insulin Resistance in HFD-Fed Mice

Subsequently, the impact of ALω-3 supplementation on insulin resistance (IR) and glucose homeostasis was assessed. An intraperitoneal glucose tolerance test (iGTT) was performed to evaluate fasting and post-glucose intake on serum glucose levels. Additionally, fasting serum glucose and insulin levels were used to calculate the homeostatic model assessment for insulin resistance (HOMA-IR). Supplementation with CMω-3, Mω-3, and ALω-3 significantly reduced the area under the curve (AUC) of the intraperitoneal glucose tolerance test (iGTT) compared to the glycerol (G) group, with reductions of 9.7% (*p* < 0.05), 12.0% (*p* < 0.01), and 17.3% (*p* < 0.001), respectively. ALω-3 supplementation showed the greatest reduction and highest statistical significance, indicating the most substantial improvement in glucose tolerance. Supplementation with Mω-3 and ALω-3 also led to lower fasting serum glucose levels (19.8% and 13.8% reductions, respectively) compared to the G group, although no significant differences were found (*p* > 0.05). Supplementation with CMω-3, Mω-3, and ALω-3 did not significantly reduce fasting insulin levels, with ALω-3 notably not increasing insulin levels, unlike the CMω-3 and Mω-3 groups, suggesting that ALω-3 may not exacerbate insulin resistance. Similar findings were observed for HOMA-IR. The potential role of omega-3 fatty acids in managing T2D remains a topic of ongoing debate. While omega-3s are hypothesized to influence signaling pathways that may alleviate complications associated with T2D [67], a meta-analysis of randomized clinical trials concluded that the evidence supporting their benefits is inconclusive, apart from improving cardiovascular risk factors in T2D [68]. Perez-Matute et al. [69] investigated the reversing effects of EPA supplementation in Wistar rats previously fed a fat-rich, hyperenergetic ‘cafeteria’ diet. Their findings reported a non-significant reduction in both plasma insulin levels and HOMA-IR following an HFD [69]. Alternatively, Claria et al. [20] evaluated the supplementation of cold-pressed MO in HFD-fed C57BL/6 mice over 12 weeks, which also showed a non-significant reduction in serum insulin levels. Dovale-Rosabal et al. [30] evaluated the effects of new molecules incorporating gallic acid into EPA/DHA-rich structured lipids (structured phenolic acylglycerols, sPAG) in HFD-fed mice. sPAG supplementation significantly decreased fasting insulin and HOMA-IR compared to sunflower oil supplementation alongside an HFD [30]. However, in our study, this effect was not observed with ALω-3 supplementation, suggesting that ALω-3 may improve glucose tolerance without directly influencing the insulin signaling pathway.

### 4.6. Effect of ALω-3 Supplementation on Hepatic and Visceral Fat Pro-Inflammatory Gene Expression in HFD-Fed Mice

Pro-inflammatory cytokines IL-6, IL-1β, and TNF-α play a crucial role in MASLD-related inflammation and progression and hepatic insulin resistance [70]. Additionally, TLR4 has been linked to inflammatory activation in the livers of obese patients, contributing to the development of hepatic steatosis, steatohepatitis, and IR [71,72]. Interestingly, the inflammatory profiles of hepatic and visceral adipose tissues are similar in obesity [73]. Therefore, treatments that attenuate inflammation in these tissues may effectively control obesity, MASLD, and IR. In this study, the relative expression of IL-6, TNF-α, and TLR4 were evaluated in the liver and visceral fat, with β-actin as the constitutive gene. The effects of different supplementation groups (CMω-3, Mω-3, and ALω-3) were also evaluated in comparison to the negative control (G). None of the supplementations (CMω-3, Mω-3, and ALω-3) significantly reduced the expression of these pro-inflammatory markers in the liver and visceral fat, except Mω-3 in the case of IL-6 expression in the visceral fat (*p* < 0.05). In the liver samples, ALω-3 reduced IL-6 expression by 6.7% compared to the G group, but this change was not statistically significant (*p* > 0.05). Mω-3 and ALω-3 also showed a trend toward decreased TNF-α expression (15.1% and 24.5% reductions, respectively) and TLR4 expression (17.5% and 31.2% reductions, respectively), although these differences were not statistically significant (*p* > 0.05). In the visceral fat, Mω-3 significantly reduced IL-6 expression by 61.8% (*p* < 0.05), while a trend toward a decrease in IL-6 was observed in the CMω-3 group (18.6%, *p* > 0.05). Mω-3 supplementation increased TNF-α expression by 6.0%, while both CMω-3 and ALω-3 reduced TNF-α expression by 10%, although these differences were not statistically significant (*p* > 0.05). Regarding TLR4 expression in the visceral fat, CMω-3 and Mω-3 showed reductions of 27.2% and 38.5%, respectively (*p* > 0.05), while ALω-3 supplementation did not display a similar pattern to that of the G group.

Histological analysis of the liver sections revealed no substantial inflammation across the groups, suggesting that the hepatic inflammatory state may not have been severe enough to detect pronounced effects from ALω-3 supplementation. When compared with the previous studies (Calder [74] and Spooner et al. [75]) on dietary supplementation and inflammation, omega-3 fatty acids stand out for their well-established anti-inflammatory properties, primarily mediated through mechanisms such as the suppression of NFκB signaling. In a study by Depner et al. [76], 16 weeks of omega-3 supplementation in an Ldlr−/− mouse model of Western diet-induced nonalcoholic steatohepatitis (NASH) significantly reduced hepatic pro-inflammatory markers, including TNF-α, TLR4, and IL-1β. Similarly, Šmid et al. [77] demonstrated that dietary enrichment with n-3 PUFAs in a high-fat methionine- and choline-deficient (MCD) diet model over six weeks led to marked reductions in hepatic expression of IL-1 and TNF-α, highlighting the potential of n-3 PUFAs to mitigate inflammation associated with non-alcoholic fatty liver disease (NAFLD). The anti-inflammatory effects of omega-3s have also been observed in adipose tissues. Perez-Matute et al. [69] and Todorčević & Hodson [78] reported reduced TNF-α expression in adipose tissue following omega-3 supplementation, corroborating their systemic effects on inflammatory markers. In addition to omega-3s, oils rich in tocopherols have demonstrated anti-inflammatory potential. Noichi et al. [79] compared T3 and α-Tocopherol in a choline-deficient, L-amino acid-defined high-fat diet (CDAHFD) mouse model of early-stage NASH over three weeks. While both isoforms reduced F4/80 expression—a marker of inflammatory cell activation—only T3 consistently suppressed inflammation, although changes in the TNF-α expression were not statistically significant [79]. Similarly, Tapia et al. [80] evaluated the effects of rosehip oil (RM), a tocopherol-rich vegetable oil, in a high-fat diet (HFD)-induced model using C57BL/6J mice over 12 weeks. RM supplementation significantly reduced TNF-α and IL-1β mRNA levels in adipose tissues compared to HFD alone, underscoring its protective role against diet-induced inflammation [80]. The trends observed in our study, especially with the Mω-3 and ALω-3 groups, suggest a potential synergy between omega-3 fatty acids and tocopherols in modulating proinflammatory markers. The combination of omega-3 with low amounts of cold-pressed maqui seed oil appeared to enhance these effects in both the liver and visceral fat, with a notable reduction in IL-6 expression in the visceral fat. While ALω-3 seems more effective in reducing liver inflammation, further studies are required to refine ALω-3 production and optimize dosage. These efforts could pave the way for the development of a nutraceutical tailored to mitigate inflammation in MetS.

### 4.7. Effect of ALω-3 Supplementation on Serum Total Antioxidant Capacity in HFD-Fed Mice

Lastly, serum total antioxidant capacity (TAC) was assessed as a comprehensive marker of systemic antioxidant defenses, reflecting the ability to neutralize free radicals and mitigate oxidative stress [81]. In this study, the G group, without antioxidant supplementation, had the lowest TAC (3.17 ± 0.1 μM). Supplementation with CMω-3 significantly increased TAC (4.81 ± 0.1 μM, *p* < 0.01), although it remained lower than other supplemented groups. Mω-3 further improved TAC (6.9 ± 0.4 μM, *p* < 0.001), and ALω-3 achieved the highest TAC (7.6 ± 0.5 μM, *p* < 0.001), surpassing all groups. These results highlight ALω-3 as the most effective antioxidant, suggesting its potential for managing oxidative stress in metabolic dysfunction. This underscores the potent antioxidant properties of MO, likely attributed to its high tocopherol and tocotrienol contents [10,48]. These findings highlight ALω-3 as the most effective antioxidant, suggesting its potential for managing oxidative stress in metabolic dysfunction and obesity-related conditions like MASLD. The observed antioxidant effects align with those reported in similar studies. For instance, Hou et al. [82] demonstrated that *Trichosanthes kirilowii* Maxim seed oil (TSO), flavonoids (FLA), and their combination (TSOFLA) enhanced serum antioxidant enzyme activity in hyperlipidemic mice. In particular, TSOFLA significantly reduced serum malondialdehyde (MDA) levels and increased superoxide dismutase (SOD) and glutathione peroxidase (GSH-Px) activity compared to an HFD-fed control group [82]. Martínez-Galán et al. [62] demonstrated that administering a high-fat diet (HFD) containing 50% lipid content in the form of structured lipids (SLs) enriched with capric acid, derived from grape seed oil, over an 8-week period led to a 24% reduction in plasma thiobarbituric acid reactive substances (TBARS), a marker of lipid peroxidation and oxidative stress, compared to mice on a standard HFD, although this difference was not statistically significant (*p* > 0.05).

### 4.8. Limitations and Future Perspectives

While the findings of this study provide valuable insights into the potential metabolic and anti-inflammatory effects of ALω-3 supplementation, several aspects warrant consideration. First, the dietary shift from a high-fat diet (HFD) to a control diet (CD) at the time of supplementation may have contributed to the observed improvements, and thus the independent effects of the ALω-3 cannot be fully isolated within the current design. Nevertheless, this approach mimics a practical dietary intervention scenario and reflects realistic nutritional transitions. Second, the duration of the intervention (3 weeks) may not fully capture longer-term metabolic outcomes, and extended supplementation periods would be useful to assess the persistence of the observed benefits. Finally, while reductions in systemic inflammation and oxidative stress were observed, the underlying molecular mechanisms were not directly evaluated in this study. Future studies incorporating mechanistic analyses and longer follow-up will help to further elucidate the role of antioxidant lipids in metabolic regulation.

Despite these limitations, this study marks a significant step forward in understanding the metabolic and inflammatory modulation by antioxidant lipids, providing a foundation for future research into their potential applications in nutritional and therapeutic strategies. Importantly, further investigations are required to characterize the positional distribution of fatty acids within the triacylglycerol backbone and to define dose–response relationships, both of which are critical for understanding structure–function associations. Although the diet-induced obesity mouse model is widely used and translationally relevant for studying metabolic alterations and MASLD, differences in the high genetic heterogeneity in human populations, along with environmental factors such as diet and lifestyle, can influence individual responses to nutraceutical supplementation, including EPA and DHA. Continued research along this line—integrating clinical trials, bioavailability studies, and mechanistic insights—will be essential to bridge the gap between preclinical findings and potential human application of antioxidant lipids as targeted nutraceuticals.

## 5. Conclusions

In this study, an antioxidant lipid (ALω-3) was synthesized via enzymatic acidolysis using non-specific lipase B from *Candida antarctica*, from a mixture of concentrated RTBO—rich in LCPUFA-n3 EPA/DHA—and cold-pressed maqui oil (*Aristotelia chilensis* (Mol.) Stuntz) in a 70/30 ratio. ALω-3 was analyzed for its fatty acid profile, its tocopherol and tocotrienol contents, and through thin-layer chromatography (TLC). Additionally, the effects of ALω-3 supplementation on body and organ weights, serum biochemical parameters, total antioxidant capacity, liver fat infiltration, and pro-inflammatory markers in the liver and visceral fat were assessed over 3 weeks in an HFD-fed reversion murine model.

The findings show that switching from a high-fat diet (HFD) to a control diet (CD) reduced body weight, with ALω-3 supplementation further improving the specific biochemical parameters. Notably, ALω-3 supplementation combined with CD significantly reduced the serum GPT levels and the area under the curve (AUC) of the intraperitoneal glucose tolerance test (iGTT), as well as increased the serum total antioxidant capacity (TAC).

The results of this study demonstrate that plasma total antioxidant capacity (TAC) in mice is directly influenced by the type of lipid supplementation administered after an HFD. The ALω-3 group showed the greatest increase in TAC (7.6 ± 0.5 μM), significantly outperforming the other groups (*p* < 0.001). This suggests that enzymatic acidolysis of concentrated omega-3 fatty acids with natural MO antioxidants in a lipid matrix synergistically enhances systemic antioxidant activity, possibly due to increased bioavailability of bioactive compounds to counteract oxidative stress under metabolic conditions.

These results suggest that ALω-3 supplementation, when combined with a dietary switch, may help reduce liver damage, glucose intolerance, and serum oxidative stress associated with MASLD and metabolic syndrome (MetS) in an HFD-fed murine model. In summary, ALω-3 demonstrated a tendency toward more favorable outcomes compared to Mω-3 (*p* > 0.05).

Finally, this study demonstrates the feasibility of using ALω-3 as a possible functional nutraceutical in dietary interventions targeting metabolic syndrome, specifically in the HFD-fed reversion murine model.

## Figures and Tables

**Table 1:** Experimental design using response surface methodology (RSM) for enzymatic acidolysis under supercritical CO_2_ conditions.

Experiment (No.)	Supercritical CO_2_ Pressure (bar)	Supercritical CO_2_ Temperature (°C)
1	100	50
2	100	80
3	300	50
4	300	80
5	200	65
6	200	65
7	200	65

**Table 2:** Primers used for amplification of inflammation-related genes in hepatic and visceral fat tissues.

Gen	Sense	Sequence	%Efficiency
β-actin (NM_007393)	FW	AGGGAAATCGTGCGTGACAT	100.1
	RV	AACCGCTCGTTGCCAATAGT	
IL-6 (DQ788722)	FW	TGATGGATGCTACCAAACTGG	100.8
	RV	TCTCTCTGAAAGACTCTGGCT	
TNFα (NC_000083)	FW	GGTGCCTATGTCTCAGCCTC	103.4
	RV	TGAGGGTCTGGGGCATAGAA	
TLR4 (NM_021297)	FW	CTGGAGAAACTGCTGCCTCA	101.5
	RV	AGCCTGGAAGAAGGTCT	

β-actin, constitutive gene. FW: Forward; RV: Reverse, IL-6: Interleukin 6, TNFα: Tumor necrosis factor-a, TLR4: Toll like receptor 4.

**Table 3:** EPA, DHA, and EPA + DHA (g/100 g TFA) contents obtained by enzymatic acidolysis under supercritical CO_2_ of belly oil from rainbow trout and cold-pressed maqui seed oil using RSM.

Experiment (No.)	EPA	DHA	EPA + DHA
1	13.07	18.38	31.45
2	12.76	20.15	32.91
3	15.07	16.89	31.96
4	16.43	22.44	38.87
5	15.27	19.55	34.82
6	15.27	19.55	34.82
7	15.27	19.55	34.82

**Table 4:** Optimization of enzymatic acidolysis process variables using supercritical CO_2_ by response surface methodology (RSM) to obtain antioxidant lipids (ALω-3).

Response Variables	Supercritical CO_2_ Pressure	Supercritical CO_2_ Temperature	Stationary Point	Optimized Values
	Part a: Optimization of the process variables			
EPA (g/100 g TFA)	300	80	Maximum	16.41
DHA (g/100 g TFA)			Maximum	22.48
EPA + DHA (g/100 g TFA)			Maximum	39.31
	Part b: Multiple response optimization of the response variables and desirability Optimized process variables to obtain ALω-3			
EPA (g/100 g TFA)	300	80	Maximum	16.43
DHA (g/100 g TFA)				22.44
EPA + DHA (g/100 g TFA)				38.87
	Part c: Experimental validation of the multiple response optimization of the response variables of Part b Optimized process variables by RSM			
EPA (g/100 g TFA)	300	80	Maximum	10.74
DHA (g/100 g TFA)				16.23
EPA + DHA (g/100 g TFA)				26.97

**Table 5:** Fatty acid profile of commercial marine omega 3 (CMω-3), mixture of LCPUFAn-3 concentrate and cold pressed maqui seed oil (Mω-3) and antioxidant lipids (ALω-3), expressed as g fatty acids (FA)·100 g^−1^ of total fatty acids (TFA).

Systematic Name	Abbreviated Name	CMω-3	Mω-3	ALω-3
Myristic acid	C14:0	N.I.	0.42 ± 0.01 ^b^	0.60 ± 0.00 ^a^
Palmitic acid	C16:0	0.53 ± 0.02 ^c^	2.92 ± 0.08 ^b^	4.16 ± 0.01 ^a^
Cis-Palmitoleic acid	C16:1n-7	N.I.	1.02 ± 0.01 ^b^	1.26 ± 0.01 ^a^
Margaric acid	C17:0	N.I.	0.17 ± 0.00	N.I.
Margaroleic acid	C17:1	N.I.	0.09 ± 0.01	N.I.
Stearic Acid	C18:0	3.43 ± 0.08 ^a^	0.60 ± 0.01 ^b^	0.54 ± 0.02 ^b^
Trans-Vaccenic acid	C18:1n-7t	N.I.	1.01 ± 0.00 ^a^	0.86 ± 0.06 ^b^
Oleic acid	C18:1n-9	5.91 ± 0.08 ^c^	10.51 ± 0.07 ^b^	13.36 ± 0.07 ^a^
Cis-Vaccenic acid	C18:1n-7	2.07 ± 0.00 ^a^	0.50 ± 0.01 ^c^	0.75 ± 0.05 ^b^
Trans-Linoleic acid	C18:2n-6t	N.I.	0.07 ± 0.00	N.I.
Cis-Linoleic acid	C18:2n-6	1.09 ± 0.02 ^c^	30.91 ± 0.10 ^b^	33.86 ± 0.09 ^a^
γ-Linolenic acid	C18:3n-6	N.I.	N.I.	0.46 ± 0.00
Eicosanoic acid	C20:0	0.51 ± 0.00 ^b^	0.08 ± 0.01 ^a^	N.I.
8- Eicosanoic acid	C20:1n-12	1.22 ± 0.03 ^a^	0.48 ± 0.00 ^c^	0.60 ± 0.00 ^b^
α-Linolenic acid	C18:3n-3	N.I.	5.37 ± 0.01 ^b^	6.34 ± 0.01 ^a^
Eicosenoic acid	C20:1n-15	0.60 ± 0.02	N.I.	N.I.
Eicosenoic acid	C20:1n-12	0.69 ± 0.02	N.I.	N.I.
Eicosenoic acid	C20:1n-9	3.65 ± 0.06 ^c^	4.46 ± 0.01 ^a^	4.02 ± 0.01 ^b^
Eicosadienoic acid	C20:2n-6	0.52 ± 0.03 ^c^	1.47 ± 0.00 ^a^	1.12 ± 0.03 ^b^
Docosanoic acid	C22:0	N.I.	0.30 ± 0.00 ^a^	0.31 ± 0.00 ^a^
Cis-11,14,17-Eicosatrienoic acid	C20:3n-3	N.I.	0.80 ± 0.00 ^a^	0.75 ± 0.02 ^b^
Erucic acid	C22:1n-1	1.87 ± 0.05	N.I.	N.I.
Docosadienoic acid	C22:2n-6	1.82 ± 0.03 ^a^	1.47 ± 0.00 ^b^	1.32 ± 0.01 ^c^
Eicosapentaenoic acid (EPA)	C20:5n-3	47.94 ± 0.36 ^a^	12.58 ± 0.06 ^b^	10.74 ± 0.13 ^c^
Docosatrienoic acid	C22:3n-3	1.72 ± 0.03 ^a^	1.01 ± 0.01 ^b^	0.80 ± 0.00 ^c^
Docosatetraenoic acid	C22:4n-3	0.66 ± 0.01 ^a^	0.34 ± 0.00 ^b^	N.I.
Docosapentaenoic acid (DPA)	C22:5n-3	3.04 ± 0.04 ^a^	2.14 ± 0.01 ^b^	1.91 ± 0.03 ^c^
Docosahexaenoic acid (DHA)	C22:6n-3	22.65 ± 0.08 ^a^	20.05 ± 0.15 ^b^	16.23 ± 0.01 ^c^
Total saturated fatty acids (TSFA)		4.47 ^c^	4.50 ^b^	5.61 ^a^
Total monounsaturated fatty acids (TMUFA)		16.01 ^c^	18.16 ^b^	20.85 ^a^
Total polyunsaturated fatty acids (TPUFA)		79.44 ^a^	76.22 ^b^	73.53 ^c^
Total fatty acids n-3 (TFA n-3)		76.01 ^a^	42.30 ^b^	36.77 ^c^

Values correspond to mean ± standard deviation (*n* = 3), N.I. = Not identified. In each column, values with different superscript letters (^a–c^) indicate significant differences (*p* < 0.05) according to one-way ANOVA test, followed by Tukey’s post hoc comparative test.

**Table 6:** Tocopherol and tocotrienol concentrations (mg/kg oil) of antioxidant lipids (ALω-3).

α-Tocopherol	β-Tocopherol	β-Tocotrienol	γ-Tocopherol	γ-Tocotrienol	δ-Tocopherol
Traces	22.9 ± 1.4 ^a^	24.9 ± 0.2 ^a^	6.8 ± 0.7 ^b^	22.9 ± 1.7 ^a^	22.4 ± 4.7 ^a^

Values correspond to mean ± standard deviation (*n* = 2). Traces: <5 mg/kg of oil. In each column, values with different superscript letters (^a,b^) indicate significant differences (*p* < 0.05) according to Tukey HSD test.

**Table 7:** Temperatures of peaks (TPeak), onset (TOnset), and endset (TEndset), according to the melting curves of reagents involved in antioxidant lipids (ALω-3) synthesis.

Sample	ΔH (J·g^−1^)	T_Onset_ (°C)	T_Endset_ (°C)	T_Peak1_ (°C)	T_Peak2_ (°C)	T_Peak3_ (°C)
LCPUFAn-3 RTBO	131.40 ± 26.52 ^ab^	−73.23 ± 0.79 ^a^	58.08 ± 0.00 ^a^	−70.30 ± 0.28 ^bc^	−42.51 ± 0.51 ^c^	-
MSO	157.10 ± 8.17 ^a^	−73.41 ± 2.79 ^a^	58.09 ± 0.01 ^a^	−71.05 ± 2.23 ^c^	−34.37 ± 0.64 ^a^	−24.07 ± 0.18 ^a^
Mω-3	111.70 ± 17.0 ^b^	−74.56 ± 0.45 ^a^	58.09 ± 0.01 ^a^	−65.93 ± 0.15 ^ab^	−46.60 ± 0.89 ^d^	−33.41 ± 0.66 ^b^
ALω-3	160.90 ± 3.41 ^a^	−75.78 ± 1.86 ^a^	58.08 ± 0.00 ^a^	−65.48 ± 2.85 ^a^	−36.44 ± 0.10 ^b^	-

Values are expressed as mean ± standard deviation (SD) (*n* = 3). In each column, values with different superscript letters (a,b,c,d) indicate significant differences (*p* < 0.05) according to ordinary one-way ANOVA (ΔH, T_Onset_, T_Endset_, T_Peak1_, and T_Peak2_) and unpaired *t*-test (T_Peak3_). Samples: Long-chain polyunsaturated omega-3 fatty acids (LCPUFAn-3) from rainbow trout belly oil (RTBO) concentrate; cold-pressed maqui seed oil (MSO); 70/30 Mixture of LCPUFAn-3 RTBO concentrate and MSO (Mω-3); and ALω-3, synthetized by enzymatic acidolysis of Mω-3 under supercritical CO_2_ conditions.

**Table 8:** Fatty liver infiltration through histological analysis. Steatosis mice score *.

Histological Feature	G	CMω-3	Mω-3	ALω-3
**Steatosis**				
Macrovesicular	0	0	0	0
Microvesicular	1	1	1	1
Hypertrophy	0	0	0	0
**Inflammation**				
Number of inflammatory foci/field	0	0	0	0
Total score	1/12	1/12	1/12	1/12

Fatty liver infiltration was evaluated through histological analysis. Male C57BL/6 mice were fed a high-fat diet (HFD) for 8 weeks and then switched to a control diet (CD) with supplementation for 3 weeks. Supplementation corresponded to glycerol (G), commercial marine omega-3 (CMω-3), a mixture of EPA/DHA concentrate and cold-pressed maqui seed oil (Mω-3), or antioxidant lipids (ALω-3) synthetized by enzymatic acidolysis of mixture under supercritical CO_2_ conditions. Steatosis was assessed by scoring macrovesicular and microvesicular fat, hypertrophy, and inflammation foci. *n* = 6 mice for each group. * Based on Liang et al. [41].

**Table 9:** Relative expression of pro-inflammatory markers in hepatic and visceral fat of high-fat diet (HFD) murine model after the different reversion treatments.

Pro-Inflammatory Marker	G	CMω-3	Mω-3	ALω-3
	Fold G			
Liver				
IL-6	1.0 ± 0.2 ^a^	1.4 ± 0.3 ^a^	1.1 ± 0.1 ^a^	0.9 ± 0.2 ^a^
TNF-α	1.0 ± 0.2 ^a^	0.8 ± 0.1 ^a^	0.8 ± 0.2 ^a^	0.8 ± 0.2 ^a^
TLR4	1.0 ± 0.1 ^a^	0.9 ± 0.1 ^a^	0.8 ± 0.1 ^a^	0.7 ± 0.1 ^a^
Visceral Fat				
IL-6	1.0 ± 0.3 ^a^	0.8 ± 0.2 ^a^	0.4 ± 0.1 ^b^	1.0 ± 0.4 ^a^
TNF-α	1.0 ± 0.1 ^a^	0.9 ± 0.2 ^a^	1.1 ± 0.3 ^a^	0.9 ± 0.2 ^a^
TLR4	1.0 ± 0.2 ^a^	0.7 ± 0.0 ^a^	0.6 ± 0.1 ^a^	0.9 ± 0.2 ^a^

Relative expression of Interleukin-6 (IL-6), Tumor Necrosis Factor-alpha (TNF-α), and Toll-like receptor 4 (TLR4) was analyzed in hepatic and visceral fat samples by qPCR. Data were normalized to the relative change with respect to control conditions (glycerol, G) and the housekeeping gene β-actin. Male C57BL/6 mice were fed a HFD for 8 weeks and then switched to a control diet (CD) with supplementation for 3 weeks. Supplementation corresponded to glycerol (G), commercial marine omega-3 (CMω-3), a mixture of EPA/DHA concentrate and cold-pressed maqui seed oil (Mω-3), or antioxidant lipids (ALω-3) synthetized by enzymatic acidolysis of the mixture under supercritical CO_2_ conditions. The mice were fasted for 6 h prior to euthanasia (*n* = 6 mice for each group). Statistical differences were determined by one-way ANOVA test, followed by Tukey’s post hoc comparative test to determine significant differences between groups. In each column, values with different superscript letters (^a, b^) indicate significant differences (*p* < 0.05).

## Data Availability

All the data are contained within the manuscript.

## Abbreviations

The following abbreviations are used in this manuscript:

MetS	Metabolic Syndrome
T2D	Type 2 diabetes
MASLD	Metabolic dysfunction-associated steatotic liver disease
NAFLD	Non-alcoholic fatty liver disease
BC	Bioactive compounds
LCPUFAn-3	Long-chain polyunsaturated fatty acids omega-3
EPA	Eicosapentaenoic acid
DHA	Docosahexaenoic acid
LA	Linoleic acid
ALA	α-linolenic acid
MAG	Monoacylglycerols
DAG	Diacylglycerols
TAG	Triacylglycerols
SCCO_2_	Supercritical carbon dioxide
GRAS	Generally Recognized As Safe
ALω-3	Antioxidant lipids
RTBO	Rainbow trout belly oil
MO	Cold-pressed maqui seed oil
CMω-3	Commercial marine Omega 3
FA	Fatty acids
Mω-3	Mixture of concentrated RTBO and MO in a 70/30 (*w*/*w*) ratio
PET	Polyethylene terephthalate
RSM	Response surface methodology
ANOVA	Analysis of variance
GLC	Gas liquid chromatography
FAME	Fatty acid methyl esters
IUPAC	International Union of Pure and Applied Chemistry
AOCS	American Oil Chemistry Society
HPLC	High-performance liquid chromatography
TLC	Thin-layer chromatography
FFA	Free fatty acids
DSC	Differential scanning calorimetry
HFD	High-fat diet
CD	Control diet
G	Glycerol
iGTT	Intraperitoneal Glucose Tolerance Test
CICUA	Institutional Animal Care and Use Committee
GPT	Glutamate pyruvate transaminase
GOT	Glutamate oxaloacetate transaminase
TG	Triglycerides
T-Chol	Total cholesterol
HDL-Chol	High-density lipoprotein cholesterol
HOMA-IR	Homeostasis model assessment of insulin resistance
IL-6	Interleukin 6
TNFα	Tumor necrosis factor-a
TLR4	Toll like receptor 4
TAC	Total antioxidant capacity
ABTS	2,2′-azino-bis(3-ethylbenzothiazoline-6-sulfonic acid)
H_2_O_2_	Hydrogen peroxide
TE	Trolox equivalents
ΔAbs	Change in absorbance
SEM	Standard error of the mean
DPA	Docosapentaenoic acid
TSFA	Total saturated fatty acids
TMUFA	Total monounsaturated fatty acids
TPUFA	Total polyunsaturated fatty acids
TFA	Total fatty acids n-3
N.I.	Not identified
H&E	Hematoxylin and Eosin
AUC	Area under curve
sAG	Structured acylglycerols
SREBP-1	Sterol Regulatory Element-Binding Protein-1
PPARγ	Peroxisome Proliferator-Activated Receptor gamma
NF-κB	Nuclear factor-kappa B
EVOO	Extra virgin olive oil
sPAG	Structured phenolic acylglycerols containing gallic acid
AHA	American Heart Association
IR	Insulin resistance
NASH	Nonalcoholic steatohepatitis
IL-1β	Interleukin 1β
MCD	Methionine- and choline-deficient
NAFLD	Non-alcoholic fatty liver disease
CDAHFD	Choline-deficient, L-amino acid-defined high-fat diet
RM	Rosehip oil
TSO	*Trichosanthes kirilowii* Maxim seed oil
FLA	Flavonoids
TSOFLA	*Trichosanthes kirilowii* Maxim seed oil + flavonoids
MDA	Serum malondialdehyde
SOD	Superoxide dismutase
GSH-Px	Glutathione peroxidase
TBARS	Thiobarbituric acid reactive substances

## Appendix A

**Table A1:** ANOVA and regression coefficients of the first-order (*Y*_1_, *Y*_2_ and *Y*_3_) and *p* values for the different response variables obtained from the enzymatic acidolysis of rainbow trout belly oil (RTBO) concentrate and cold-pressed maqui seed oil (MO) under supercritical CO_2_.

Process Variables	Response Variables					
	(*Y*_1_) EPA		(*Y*_2_) DHA		(*Y*_3_) EPA + DHA	
	Coefficient	*p* Value	Coefficient	*p* Value	Coefficient	*p* Value
Constant	8.07		23.17		34.85	
A	0.00	0.52	−0.02	0.00	−0.04	0.01
B	0.09	0.02	−0.11	0.01	−0.07	0.03
A × B	-	-	0.00	0.00	−0.00	0.04
R^2^	79.04		99.93		95.18	
Adjusted R^2^	68.57		99.86		90.36	
SE	0.74		0.06		0.77	
MAE	0.47		0.04		0.50	
DW value	0.75		0.58		0.58	

Abbreviations: R^2^ (regression coefficient), SE (standard error), MAE (mean absolute error), DW (Durbin–Watson), and RA (residual autocorrelation). Process variables: A (Supercritical CO_2_ Pressure, bat), B (Supercritical CO_2_ Temperature, °C). Responses variables: (Y_1_) EPA, (Y_2_) DHA, (Y_3_) EPA + DHA.
